# A Touch Enabled Hemodynamic and Metabolic Monitor

**DOI:** 10.1002/advs.202502138

**Published:** 2025-04-17

**Authors:** Omeed Djassemi, Tamoghna Saha, Ponnusamy Nandhakumar, Muhammad Inam Khan, Hannah Fishman, Sara Earney, Chochanon Moonla, Yuchen Xu, Henry Thai, Sofia Morales‐Fermin, Gyeongho Kim, Rhea Park, Beya Acot, Oscar Wu, Cannon Wurster, An‐Yi Chang, Christopher Cheung, Julia Silberman, Shichao Ding, Joseph Wang

**Affiliations:** ^1^ Aiiso Yufeng Li Family Department of Chemical and Nanoengineering University of California San Diego La Jolla CA 92093 USA; ^2^ Shu Chien‐Gene Lay Department of Bioengineering University of California San Diego La Jolla CA 92093 USA; ^3^ Department of Mechanical and Aerospace Engineering University of California San Diego La Jolla CA 92093 USA; ^4^ Department of Electrical and Computer Engineering University of California San Diego La Jolla CA 92092 USA

**Keywords:** blood pressure, hormones, metabolites, sweat, touch sensing

## Abstract

Accurate health analysis demands real‐time tracking of multiple biomarkers and vital signs under dynamic physiological conditions. Current multimodal hybrid platforms provide biochemical and biophysical data but are limited by active sweat collection for biochemical sensing and bulky designs for biophysical sensing. Here a touch‐enabled platform is presented that simultaneously monitors vitals and metabolic markers. With a simple tri‐finger touch, the platform measures mean arterial pressure and heart rate using photoplethysmography, and glucose, uric acid, and cortisol at rest by leveraging the natural perspiration at the fingertip. Extended studies involving diverse activities reveal strong dynamic interplay among the metabolic and vital profiles, with mean arterial pressure showing the highest sensitivity to cortisol fluctuations. The platform delivers comprehensive health information linking diet, lifestyle, metabolism, and serves as an early metabolic or hormonal stress indicator. Valuable insights gained through the platform position it as a promising tool for personalized health and wellness management.

## Graphical abstract

This work presents a touch‐based hybrid platform for simultaneous monitoring of vital signs and sweat metabolites. With a simple tri‐finger touch of 4–6 min, users can concurrently get to know their glucose, uric acid, cortisol levels from fingertip sweat, along with blood pressure and heart rate. Measuring both biophysical and biochemical signatures from a single platform allows comprehensive health information acquisition linking diet, lifestyle, and stress conditions, and can serve as a promising tool for personalized health and wellness management.

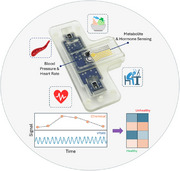

## 1 Introduction

Merging chemical and biophysical sensing modalities into a single form factor is paving the way for comprehensive and deeper understanding of human physiology.^[^ ^1^, ^2^, ^3^, ^4^ ^]^ This coupling, referred interchangeably as “*multi‐modal*” or “*hybrid*”, can be deployed on‐body as either wearables or as a point‐of‐care (POC) device.^[^ ^3^, ^4^, ^5^ ^]^ These platforms hold distinct advantages: a) eliminate the burden of having multiple individual sensors placed on the body; b) reduce device related expenditures; c) are crucial for dynamic health tracking under diverse daily activities and settings and d) hold great clinical relevance, especially for subjects suffering from serious medical conditions, such as hypo/hyperglycemia, hypertension, diabetes related stroke, cardiac diseases, retinopathy, nephropathy, septic shock, and infectious diseases like COVID 19.^[^ ^6^, ^7^ ^]^ Recent advancements in nanotechnology, material science, and polymer chemistry, have enabled multi‐modal platforms to comprise of soft and flexible components, allowing their conformal attachment on skin and non‐invasive operation with minimal user discomfort.^[^ ^2^, ^3^, ^8^ ^]^ These platforms can thus be applied on a wide range of populations – from neonates to elderly.^[^ ^9^ ^]^ Furthermore, advanced wireless technologies embedded into these platforms facilitate real‐time monitoring and data storage in mobile devices to make them directly accessible to medical professionals through cloud database.^[^ ^10^, ^11^ ^]^ Hence, hybrid sensing modules deliver simultaneous multi‐source information that leads to greatly improved prediction of health trends, disorders, and diagnostic outcomes – useful for both healthy and vulnerable populations.

Amongst different vital signs, real‐time measurement of blood pressure (BP) have garnered tremendous interest for several decades.^[^ ^12^, ^13^ ^]^ BP is one of the most important parameters for assessing the risk mortality due to its close association with diverse medical conditions such as pulmonary hypertension/hypotension, arrhythmia, pericardial diseases, kidney diseases, and diabetes.^[^ ^14^ ^]^ Frequent, convenient and accurate BP measurements are thus critical for the management of different medical conditions. Current clinical BP measurements are carried out through sphygmomanometer, arterial catheterization (AC), tonometry, and ultrasound wall tracking, in which AC and tonometry are highly operator dependent, time consuming (for locating the correct artery for measurement), and unsuitable for extended use.^[^ ^15^, ^16^ ^]^ Recent studies have shown considerable potential of wearable ultrasound imaging devices for BP analysis.^[^ ^3^, ^17^, ^18^ ^]^ However, intensive fabrication strategies, power consumption concerns, and tethered operation during on‐body analysis, make these devices less scalable and simplistic for long‐term operation. Sphygmomanometers are commercially available (cuff BP monitors) that temporarily obstruct blood flow and generate only a single BP reading per measurement.^[^ ^19^ ^]^ In contrast, photoplethysmography (PPG) is a touch‐based, non‐invasive, rapid, continuous, and user friendly hemodynamic measurement technique that can deliver BP information through time‐series waveform analysis.^[^ ^20^ ^]^ PPG signal acquisition depends on combinations of infrared (IR) light, red, and or green light (between a source and receiver) to measure the volumetric variations of blood circulation.^[^ ^21^, ^22^ ^]^ Moreover, PPG platforms are integrable, consume low power (few milliwatts), and low‐cost alternatives. Generally, PPG sensors use principles of pulse‐wave analysis (PWA), pulse transit time (PTT) and pulse‐wave velocity (PWV) to calculate systolic and diastolic pressures.^[^ ^17^, ^21^ ^]^ Despite these advances, there have been limited studies on hybrid BP‐chemical platforms – with no studies integrating optical BP with metabolic monitoring.

Epidermal metabolic monitoring with maximal user comfort is mostly conducted with either interstitial fluid (ISF) or sweat, since these biofluids are easily accessible and their biomarker levels have shown good correlation with their levels in blood.^[^ ^23^, ^24^, ^25^, ^26^, ^27^, ^28^, ^29^ ^]^ ISF sensing is possible either with microneedles (MNs) or through reverse iontophoresis (RI).^[^ ^24^, ^30^, ^31^, ^32^, ^33^ ^]^ MNs need complex fabrication strategies, while RI increases the chances of skin irritation from electric field.^[^ ^34^ ^]^ Although sweat based hybrid platforms exist, they either function under physical exertion or with iontophoresis – making them less ergonomic under daily usage, especially for the geriatric, obese or neonate populations.^[^ ^3^, ^4^, ^35^ ^]^ Fingertip sweat can potentially address these issues, considering its high sweat gland density possession (≈400 glands per cm^2^).^[^ ^23^, ^36^, ^37^ ^]^ This leads to a sweat generation rate of ≈50–100 nl min^−1^, which is sufficient to conduct electrochemical sensing at rest.^[^ ^23^, ^38^ ^]^ Leveraging the high sweat rate at the fingertips, our group and several other teams have developed a rapid and simple fingertip touch‐based sweat sensing devices for a wide variety of key biomarkers.^[^ ^39^, ^40^, ^41^, ^42^, ^43^, ^44^ ^]^ These platforms consist of a functionalized electrochemical transducer and a porous hydrogel. The hydrogel stays interfaced on the sensor and upon touching, sweat biomarkers get transported from the fingertip to the sensor through the hydrogel via diffusion and Laplace pressure. Such fingertip chemical sensing operation is completely non‐invasive, exertion‐free with maximum user comfort, with the measured sweat biomarkers showing good correlation with their levels in blood.^[^ ^39^ ^]^ Despite the ease of operation and promise, touch‐based hybrid multimodal chem‐physical sensing platforms have not been realized.

**Figure 1:**
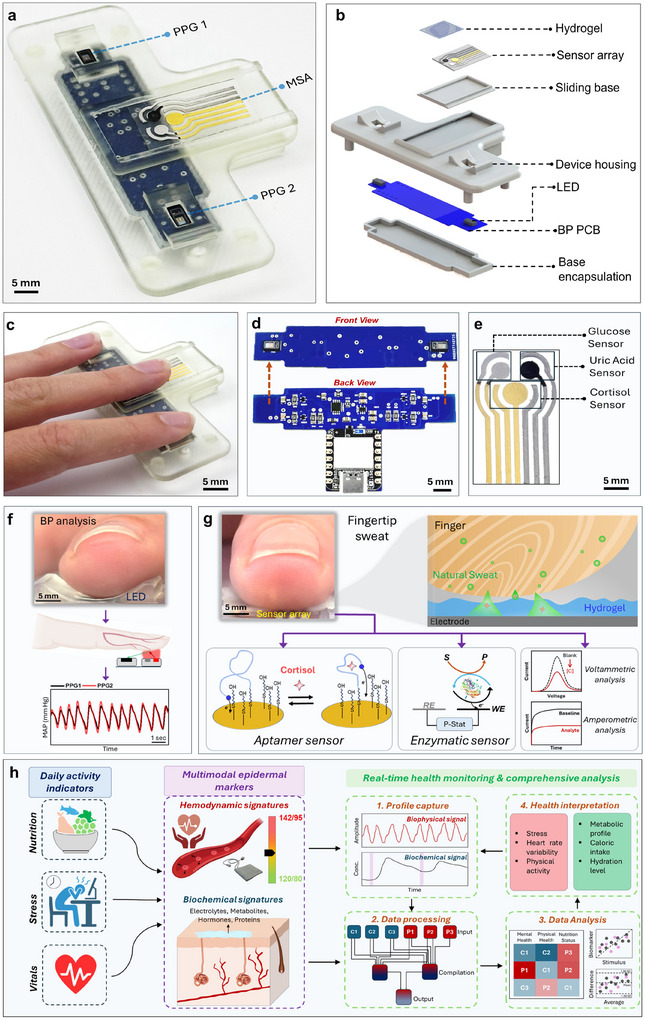
Details of TEMPT platform. a) Optical image of TEMPT highlighting the BP circuitry and MSA inside a 3‐D printed casing. b) Blown out schematic showing all the individual components of TEMPT. c) Image showing how all three fingers conformally sit on TEMPT for simultaneous BP and metabolic monitoring. d) BP board highlighting the two PPG sensors in the front view and the associated circuitry in the rear view. e) MSA of seven electrodes comprising both screen‐printed, and metal‐sputtered working electrodes for biosensing of glucose, uric acid, and cortisol (and corresponding reference and counter electrodes). f) Image showing how the fingertip conformally sits over the PPG sensor holder slot. IR light emitted by the PPG LEDs passes through skin for time series analysis for BP estimation. g) The fingertip also conformally sits over the hydrogel on MSA. The hydrogel drives the biomarker transport due to natural perspiration from the fingertip to electrochemical sensor array. MSA relies on both aptamer and enzymatic recognition reactions. h) TEMPT captures hemodynamic and metabolic data of an individual during routine daily activities. Processing and analyzing the data will allow comprehending how much and to what extent does each signature contributes to the health wellness of an individual under various physiological conditions. The resulting multi‐source chem‐physical data is processed and fused to offer deeper understanding of a user's health toward improved decision making in lifestyle.

Obtaining comprehensive health information requires detailed understanding of to what extent each bio‐signal contributes to the wellbeing on a regular basis. In pursuit of this objective, we introduce here an integrated touch‐enabled metabolic and physiological tester (TEMPT) platform, that enables concurrent, convenient and non‐invasive monitoring of BP, heart rate (HR) and several sweat metabolites. Using a simplistic tri‐finger touch approach, TEMPT merges BP and chemical sensing on a single form‐factor to capture metabolic and hemodynamic data of an individual during routine daily activities. It has two components – a BP circuit board and metabolic sensor array (MSA) all encased in a single 3D printed housing (**Figure** **1**a; Figure S1; Video S1, Supporting Information). The BP board has two PPG sensors on each end and is protected by a 3D printed base case, as shown in Figure 1b. Another 3D printed housing with two openings sits on top of the BP board. These openings allow the emitted light from light emitting diodes (LEDs) to pass through a fingertip skin for obtaining PPG waveforms. This housing also contains a slot for the MSA that rests on a sliding base to allow dynamic adjustment of the fingertip (considering interpersonal girth and length variations) with the hydrogel and MSA upon touch. The TEMPT's design thus allows the user to use three fingers (index – BP, middle – metabolic sensing, and ring – BP) simultaneously to generate their multi‐source metabolic‐hemodynamic health information (Figure 1c).

Such touch‐enabled multiplexed hybrid sensing platforms can carry out simultaneous monitoring of several key metabolic and physiological parameters. Overall, TEMPT is lightweight (≈20 g), portable, and ergonomic, supporting user‐friendly, rapid, and effortless operation. The BP circuit board is T‐shaped and is developed on a standard FR‐4 plate (Figure 1d). The front side hosts two‐time synchronized PPG sensors for enabling the time series analysis of BP prediction (Table S1, Supporting Information). The electrochemical‐based MSA module comprises of both screen printed and metal sputtered electrodes on a polyethylene terephthalate glycol substrate (PETG) (Figure 1e). In this work, we have designed TEMPT as a cardiac wellness and nutrition tracking tool. Our MSA comprises glucose, uric acid (UA) and cortisol sensors, due to their close association with BP (Figure S2, Supporting Information). The fingertip conformally rests on the holder slots for simultaneous BP and metabolic monitoring (Figure 1f,g). Two‐time synchronized PPG sensors emit IR light onto the fingertip to capture the volumetric changes in blood flow and to conduct time series analysis for BP prediction (Figure 1f). The MSA functions on natural perspiration, in which the PVA hydrogel placed over the MSA facilitates transport of sweat biomarkers from the fingertip to the electrodes upon touch (Figure 1g). It also establishes a soft and conformal interface for the fingertip.^[^ ^45^ ^]^ The electrodes function with different bioreceptors and detection modes toward specific diagnostic applications. The present MSA involves both aptamer (cortisol) and enzymatic (glucose and UA) biosensors in connection to voltametric and amperometric signal transductions, respectively. Overall, TEMPT is the first demonstration of a touch‐based hybrid multi‐modal system, which is simple and convenient to use frequently with minimal discomfort (Video S2, Supporting Information). It enables dynamic tracking of the user's response to diverse daily activities and health conditions, such as nutritional, cardiac wellness and stress levels, useful toward maintaining a healthy lifestyle (Figure 1h). Compiled and comprehensive analysis of biochemical and biophysical data can assess the multi‐source health data and assist with dynamic tracking and prediction of diverse abnormalities, like metabolic syndromes, impact of stress on metabolism, obesity and insulin resistance, respiratory distress, gout‐diabetes co‐management, and inflammation/auto immune disorders. The versatility of the TEMPT platform allows customizing and tailoring the target biomarkers and vital signs to specific user cases and their corresponding diagnostic requirements. Hence, such touch‐based, non‐invasive technologies can be a useful addition to the group of next‐generation stress‐free multimodal sensing systems. Its speed, simplicity and accuracy hold considerable potential for frequent self‐testing health assessment toward short‐sample‐to‐answer times at points‐of‐need.

## 2 Results

### 2.1 Optical BP Monitoring System

**Figure 2:**
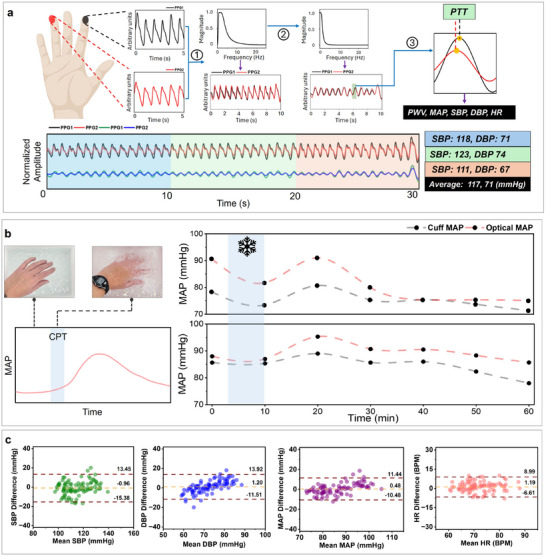
Optical BP system. a) Schematic showing how the raw PPG waveforms from the fingertip upon touch is processed to derive PWV, MAP, SBP, DBP, and HR. Our algorithm captures PPG data for 30 s to generate SBP and DBP value every 10 s. The average value from every 10 s is reported. PPG waveforms after steps 1 and 2 are plotted across a 30 s measurement. Blue arrow: Sequential steps b) Schematic showing the CPT test protocol (dipping hand in ice bath) and the obtained MAP profile from two subjects. Blue zone: CPT duration. MAP increased due to vasoconstriction, which is an established outcome of CPT. Predicted MAP profile in TEMPT stayed in close agreement with cuff data. c) Bland‐Altman plots for SBP, DBP, MAP, and HR analysis versus cuff. Purple dashed line: ±1.96.SD (95% confidence interval (CI)), yellow dashed line: bias. The majority of data points lay in the 95% CI with an overall error ranging 6–10 mm Hg versus cuff values.

PPG has recently gained traction as a continuous, non‐invasive, and cuff‐free BP monitoring technique.^[^ ^46^ ^]^ PPG estimates BP with either machine learning algorithms, pulse transit time (PTT) derived from combined electrocardiography (ECG) and PPG data, or through pulse wave velocity (PWV) correlations. PWV is a newer approach in the class time‐series analysis methods for BP estimation.^[^ ^47^, ^48^ ^]^ The PWV principle relies on using the speed at which the blood's pulse wave travels through the arteries. The mathematical relationship can be established by calculating the PTT of the systolic peak of a pulse‐waveform at two distinct locations (**Figure** **2**a).

(1) $$PWV=\frac{L}{\Delta t}$$

where, *L* is the distance between two‐time synchronized PPG sensors and Δ*t* is the time difference between the systolic peaks of two time‐synchronized PPG waveforms.^[^ ^15^ ^]^ The fingers are an optimal site for PPG data acquisition due to their rich vascularization, high signal quality, and glabrous skin. These factors, combined with the ease of maintaining consistent contact pressure, makes finger a reliable and effective location for PPG‐based health monitoring.^[^ ^49^, ^50^ ^]^ Several stages of digital signal processing are used to output all vital sign information from the PTT (Figure 2a). Initially, the acquired raw infrared PPG signal from two fingers gets passed through a second order Butterworth bandpass filter with cutoff frequencies of 0.5 – 3.5 Hz (step 1). The IR waveform from both PPG sensors show the best correlation amongst each other versus red and green light (Figure S3, Supporting Information). The filter range removes noise and DC drifts in the waveforms. Next, a third order Butterworth lowpass filter with a cutoff frequency of 0.05 Hz gets applied to completely smoothen the time‐synchronized PPG waveforms for pulse‐wave analysis (step 2). Although infinite pulse response has been used to deconvolute PPG waveforms, the Butterworth filter (BF) effectively captures the human heartbeat frequency (0.8–3 Hz, corresponding to 48–180 beats per minute (bpm)) by effectively retaining relevant heart rate information.^[^ ^51^ ^]^ Also, BF has a maximally flat frequency response in the passband, which ensures smoother transition between frequencies (vs Chebyshev filter class). After the final signal processing step, the average PTT is determined and the PWV is calculated (step 3) (Figures S4 and S5, Supporting Information). The PWV is fitted into a second order polynomial equation for systolic blood pressure (SBP), diastolic blood pressure (DBP), and mean arterial pressure (MAP) estimation, which is calculated as follows:

(2) $$MAP=DBP+\frac{1}{3}\left(SBP-DBP\right)$$

Further details on the second‐order polynomial used to estimate SBP and DBP can be found in Text S1 (Supporting Information). The comparison of the measured BP with literature can be found in Table S2 (Supporting Information). To maximize output and improve probabilistic outcomes of BP in correlation to the gold‐standard cuff, data was recorded for 30 s to generate three sets (every 10 s) of unique BP values. We used the average value as the BP output, as shown in Figure 2a. The ring and index fingers were utilized for the PPG acquisition in TEMPT, as other finger combinations (varying distance between two PPG sensors) did not generate reliable trends (Figure 1c; Figure S6, Supporting Information). The consistency of the pressure applied by utilizing two fingers onto the optical sensors versus one is also essential for accurate estimations. To further study the intersubject variations on this phenomenon, we closely examined the adequate force necessary to give maximum PPG signal fidelity (Text S2; Figures S7 and S8, Supporting Information). The ideal pressure necessary to be applied on the sensors was found to be 5.7 ± 0.08 kPa. Contamination on the fingertip (like oil or dirt) does allow IR light to penetrate the skin and generate a PPG waveform, but with lesser accuracy (Figure S9, Supporting Information). Insignificant changes in the PPG waveform were also observed with varying skin tones (Figure S10, Supporting Information). Similar PPG waveforms with a higher increasing rate of MAP and HR were observed during exercise (Figure S11, Supporting Information).

The innate dynamics of human BP and PWV‐based BP algorithm were tested through a cold pressor test (CPT) experiment, as shown in Figure 2b. The BP response to CPT test is a reproducible and stable characteristic in the general population.^[^ ^52^ ^]^ After baseline BP measurements, subjects submerged their hand in an ice‐bath for 5 min. Both subjects showed an increase in MAP, signifying that PTT can fluctuate during CPT (Figure 2b). The cold temperature causes vasoconstriction to increase vascular resistance. The arteries become stiffer and narrower during this short window, leading to faster propagation of the pulse wave and increased PWV to elevate the MAP.^[^ ^53^, ^54^ ^]^ PTT decreases due to its inverse relationship with PWV. Statistical analyses of SBP, DBP, MAP, and HR are demonstrated via Bland‐Altman plots (Figure 2c) to better understand the agreement between the values of cuff and the TEMPT platform. The majority of measured data points (N = 10 subjects, 90 data points) lies within the 95% confidence interval (CI, ±1.96SD (standard deviation)). The mean absolute error (MAE) of SBP, DBP, MAP, and HR range 5.69, 5.00, 4.33, and 3.26 mmHg, respectively, while the bias ranges 0.96, 1.20, 0.48, and 1.19‐mm Hg, respectively. Our system's low MAE matches the MAE of other PPG‐based BP devices, and error is also below the standards (<15 mm Hg) set by the U.S. Food and Drug Administration and Advancement of Medical Instrumentation for clinical grade BP predicting instruments.^[^ ^16^, ^55^ ^]^

### 2.2 Metabolic Sensor Array System

**Figure 3:**
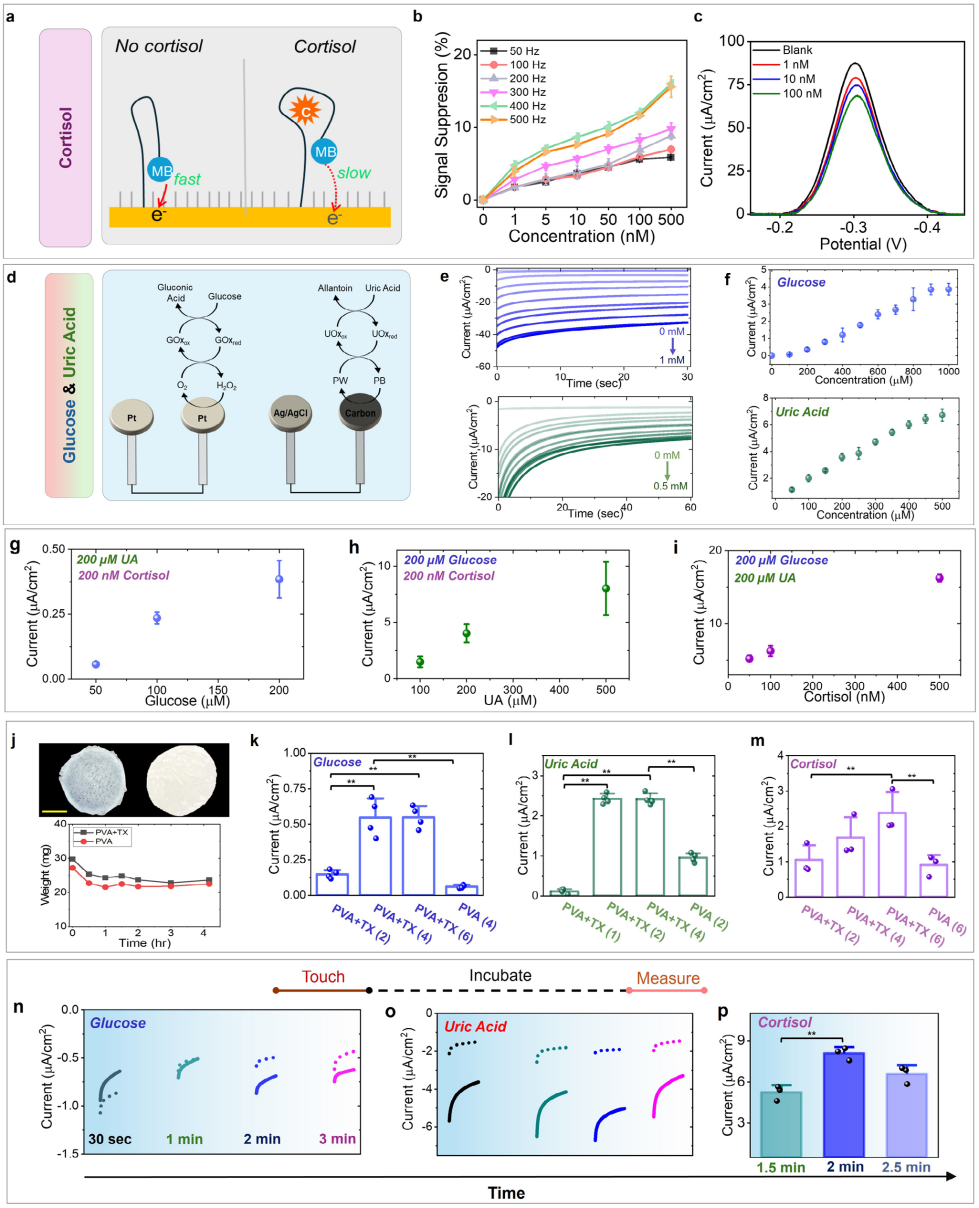
Analytical performance of metabolic sensors. a) Schematic showing the “signal‐off” based working mechanism of cortisol aptamer. b) Frequency optimization of the aptamer sensor to achieve the highest signal change with varying cortisol concentrations. c) Response of the aptamer sensor under lower cortisol concentrations, illustrating the ability to differentiate between blank, 1, 10, and 100 nm. d) Schematic showing the working mechanism of first‐generation Pt‐based glucose and PB‐based UA sensors. e) Chronoamperometric response of the glucose and UA sensors for successive 100 and 50 µm glucose and UA increments, respectively. using an applied potential of −0.25 and −0.3 V, respectively. using an applied potential of −0.25 and −0.3 V, f) Calibration plots of glucose and UA sensors highlighting their wide linear dynamic range. Error bars denote SD from n = 3. Cross talk studies for each metabolite, showing minimal interference on (g) glucose under 200 µm UA and 200 nm cortisol, h) UA under 200 µm glucose and 200 nm cortisol, and i) cortisol under 200 µm glucose and 200 µm UA. Error bars denote SD from n = 3. j) Optical images and swelling trends of porous and non‐porous PVA hydrogel in 1X PBS. k‐m) In‐vitro optimization of analyte diffusion time through the hydrogel. Results show that 2 µL solutions containing physiological concentration of glucose, UA, and cortisol take 4, 2, and 6 min, respectively, to generate maximum signal change. TX refers to Triton‐X and the number in parathesis denotes time in minutes. **p < 0.05 from two‐sample t‐test. Error bars denote SD. n‐p) Investigation of fingertip touch time duration with MSA and hydrogel. A touch time of at least 2 min is needed to transfer all the natural perspiration secreted fingertip glucose and UA to the hydrogel. A 2 min touch time is needed to transfer all the fingertip sweat cortisol. Dotted line: blank response; Solid line: response from metabolite. Error bars denote SD from n = 3.

The MSA consists of three electrochemical transducers modified with different bioreceptors to simultaneously measure multiple biomarkers from a single fingertip touch. This electrochemical module can be customized for specific sensing applications by modifying the transducer with different bioreceptors. In this study, the MSA has been used for simultaneous measurements of cortisol, UA, and glucose using aptamer, uricase and glucose oxidase enzymes, respectively. The cortisol sensor relies on an aptamer bioreceptor containing a methylene blue (MB) redox reporter, which undergoes a conformational change upon specific binding with the target cortisol. Our aptamer follows a *“signal‐off”* mechanism, where the electron transport rate decreases upon increasing the cortisol concentration (**Figure** **3**a). This is quantified by tracking the MB reduction current using square wave voltammetry (SWV). Figure 3b shows that a SWV frequency of at 400 Hz leads to the highest signal diminution with cortisol in‐vitro. The sensor can readily differentiate between blank, 1, 10, and 100 nM cortisol (net current density changes by ≈20 µ*A*/cm^2^ in 1X PBS solution, with a limit of detection (LOD) of 750 pM) at ‐0.3 V (Figure 3c). Such sensitivity justifies its practical applicability for measuring cortisol in sweat, where the concentration ranges ≈1–20 nM.^[^ ^56^ ^]^ Both glucose and UA enzymatic sensors are first generation based (where H_2_O_2_ reduction current varies proportionally to biomarker concentration) (Figure 3d). The glucose sensor validation is shown till 1 mM with 100 µM increments at −0.25 V, while UA sensor validation is shown till 500 µM with 50 µM increments at −0.3 V in‐vitro in 1X PBS (Figure 3e). This high linear dynamic range ensures that our sensors can certainly detect the physiological range in sweat.^[^ ^57^, ^58^ ^]^ Based on this, the achieved sensitivities ranged ≈$4.21\;\frac{\mu A}{cm^{2}.mM}$ and ≈$15\;\frac{\mu A}{cm^{2}.mM}$ for glucose and UA sensor, respectively (Figure 3f). Other in‐vitro electrochemical characterization studies (such as selectivity, stability, sensor‐to‐sensor response) of the sensors are shown in Figures S12–S14 (Supporting Information). The crosstalk interference of each sensor was also evaluated in‐vitro. The concentration of one metabolite was varied by keeping the other two metabolite concentrations constant in (Figure 3g–i). The acquired response was like the trends observed in Figure 3e,f, indicating minimal inter‐metabolite interferences. The reusability of the different sensors further enhances their practical utility, as illustrated in Figure S12 (Supporting Information). Other relevant sweat analytes also showed minimal interference on the respective sensors (Figures S13–S15, Supporting Information). Their performance metrics is shown in Tables S3–S5 (Supporting Information).

Next, the sensor performance was evaluated with hydrogel. We used a soft and porous hydrogel disk in the MSA to transport sweat from the fingertip to the electrode array (Figure 3j). A porous hydrogel will facilitate analyte transport due to the additional capillarity effect, alongside diffusion.^[^ ^59^ ^]^ It will also mitigate the effects of skin properties (like dry/oily) on the sensor response and can be reused after soaking in buffer. Our hydrogel was polyvinyl alcohol (PVA) based, and its dense porous network was created using micelle templating with Triton‐X (Figure S16, Supporting Information). Long‐term swelling studies were also conducted where the porous hydrogel did not show any significant swelling in 1X PBS, supporting minimal analyte retention in the hydrogel matrix (Figure 3j).

Subsequently, the response time of all three sensors was evaluated in the presence of the hydrogel. First, the analyte diffusion time was investigated in‐vitro. 2 µLs of 150 µm glucose solution dropped over the porous hydrogel covering the glucose sensor showed maximum response after 4 min (p<0.05 vs 2 min diffusion time) (Figure 3k). The non‐porous PVA counterpart showed sixfold slower response after a similar incubation time. The UA sensor showed maximum signal change after 2 min, post dropping 2µLs of 200 µm UA solution. The magnitude of the change was ≈25 times and ≈2.5 times greater versus 1 min incubation time and non‐porous PVA gel (2 min incubation), respectively (Figure 3l). The cortisol sensor showed maximum signal change after 6 min, post dropping 2µLs of 5 nm cortisol solution (Figure 3m). This change was also ≈2.5 times higher than the non‐porous PVA gel's response with similar incubation time. Based on the low hydrogel thickness, the diffusion coefficients of glucose, UA, and cortisol in the porous PVA gel were measured to be ≈1.17  × 10^−5^, 2.34  × 10^−5^, 7.81  × 10^−6^ cm^2^ s^−1^, respectively (Figure S17, Supporting Information).

The duration of the touch time on the MSA was subsequently investigated. The fingertip was cleaned and 2 µL of 150 µm glucose, 200 µm uric acid, and 5 nm cortisol solutions were applied onto the index fingertip separately before testing. The response was captured by touching the hydrogel on the MSA and by following the incubation timelines, as determined from Figure 3k–m. Both glucose and UA sensors showed maximum signal change after 2 min of touching, while maximum signal suppression occurred after 2 min of touch with the cortisol sensor (Figure 3n–p). Overall, by comparing Figure 3j–p, it can be seen that: a) the catalytic activity of glucose on Pt is relatively slower than UA on PB carbon electrode (similar molecular weight and touch time, but varying diffusion timelines). b) Although cortisol is heavier (≈2x) than glucose and UA, its lower physiological concentration in sweat is the major contributing factor toward the longer incubation time in gel (Figure 3m). Subsequent in vitro and in vivo SWV cortisol measurements are further illustrated and discussed in Figures S18 and S19 (Supporting Information). Finally, the effect of the touch pressure on the sensor response was evaluated (Figures S20–S22, Supporting Information). The ideal applied pressure needed to get the optimal sensor response ranged ≈5–6 kPa, in which the glucose sensor was found to be more sensitive to pressure changes than UA sensor (Figures S21 and S22, Supporting Information). This effect of applied pressure or other external movements can be simply accounted by multiplying the response with a correction factor, as explained in Figure S23 (Supporting Information).

### 2.3 On‐Body Performance of TEMPT

**Figure 4:**
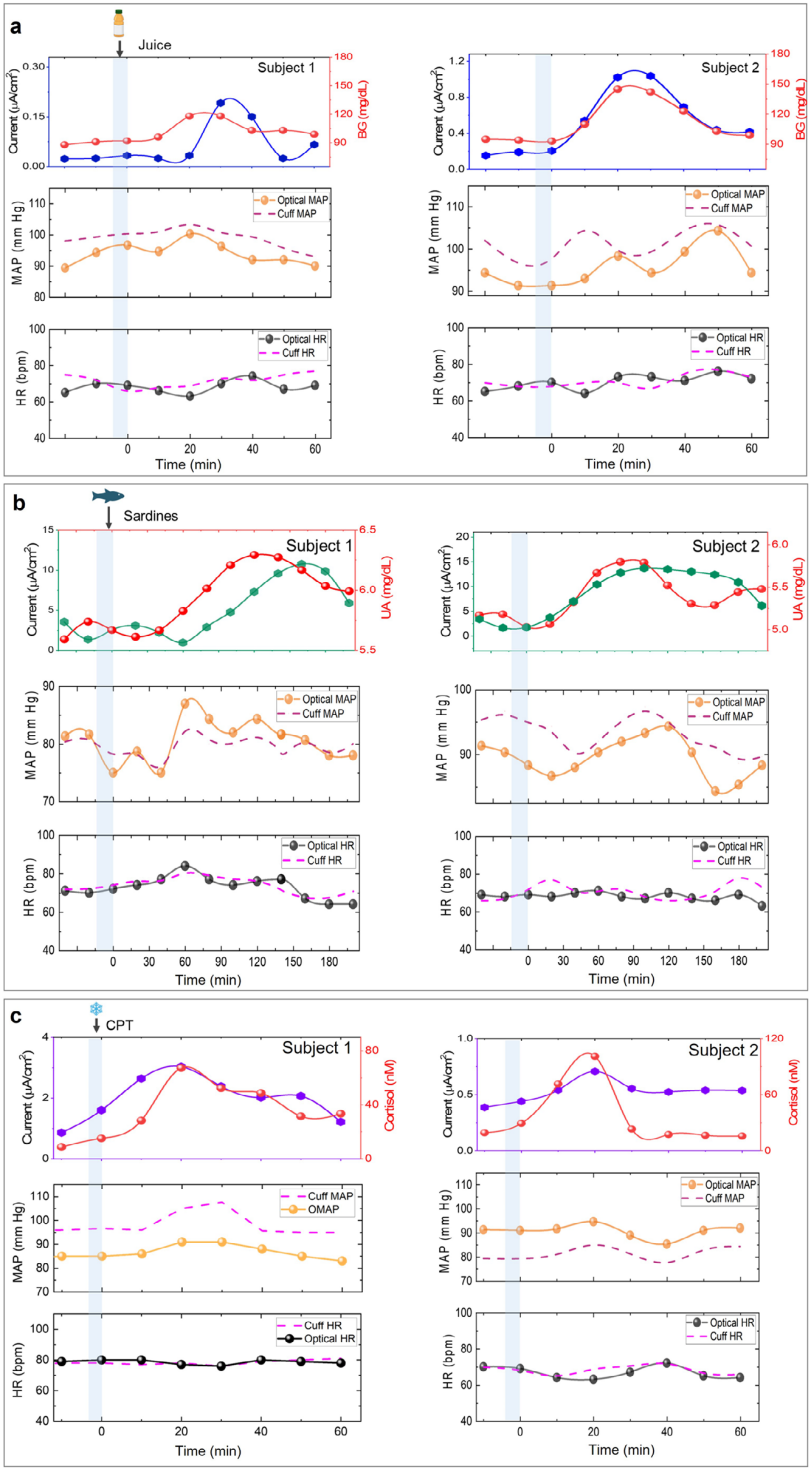
On‐body performance of metabolic sensors of TEMPT a) Plots from two subjects showing the temporal profile of sweat glucose, blood glucose, MAP, and HR with meal intake. The glucose sensor testing protocol involves 2 min of touch and 4 min of incubation before measurement. b) Plots from two subjects showing the temporal profiles of sweat uric acid, blood uric acid, MAP, and HR upon sardine consumption. The uric acid sensor testing protocol involves 2 min of touch and 2 min of incubation before measurement. c) Plots from two subjects showing the temporal profiles of sweat cortisol (TEMPT), osmotically extracted sweat cortisol (from immunoassay), MAP, and HR with CPT test. The cortisol sensor testing protocol involves touch and incubation times of 1.5 and 6 min, respectively before measurement.

The TEMPT platform underwent extensive individual assessment of the metabolites and vital signs on multiple healthy subjects, with frequent repeated touches yielding distinct vital‐sign and metabolic temporal profiles. All tests were performed at rest since the entire purpose was to make the TEMPT work under minimal user discomfort. Glucose was evaluated first as its levels play a crucial role toward adjusting the medication, diet, and lifestyle in diabetic individuals. High prevalence of hyperglycemia and hypertension is common in diabetes, with significant connections to increased age, and overweight/obesity.^[^ ^60^, ^61^ ^]^ Hypoglycemia can cause symptoms like hunger, increased HR, dizziness, confusion, sweating, and fainting.^[^ ^62^ ^]^ Thus, understanding glucose, BP, and HR dynamics is essential to manage dietary habits, cardiovascular health, and medications, which we have investigated in **Figure** **4**a. The touch, incubation, and measurement timelines were selected from Figure 3k–p and the subjects effectively applied the same amount of pressure every time during touch. Both subjects showed a net current change of ≈0.16 and ≈0.7 µ*A*/cm^2^in glucose sensor with juice consumption (Figure 4a). This change corelates to ≈200–300 µm glucose (Figure 3b), which is the established average glucose concentration in sweat.^[^ ^23^ ^]^ This change also correlated well to their respective BG concentration change (≈16 *mg*/*dL* for subject 1 and ≈42 *mg*/*dL* for subject 2). Moreover, the timeline of maximum sweat glucose change lagged by ≈10 min to BG peak in subject 1. The MAP and HR profiles across both subjects showed low fluctuation and deviation from their respective measurement techniques (TEMPT platform and cuff). The average deviation of the cuff MAP and HR ranged ≈3 mmHg and ≈3 bpm, respectively, while the average deviation of TEMPT's MAP and HR ranged ≈4 mmHg and ≈3 bpm respectively. Such low variations in the MAP and HR are plausible in this experimental scenario, as early studies have indicated that moderate consumption of fruit juice has minimal effect on BP.^[^ ^63^, ^64^ ^]^

Next, the relationship between BP and UA was investigated (Figure 4b). Hyperuricemia is prevalent in hypertensive patients and is considered a risk factor in cardiovascular diseases.^[^ ^65^, ^66^ ^]^ Also, higher UA production is even associated with oxidative stress and inflammation, which can also elevate the BP.^[^ ^67^ ^]^ Figure 4b investigates the temporal relationship between UA and vitals for 200 min. After acquiring baseline response, the subjects consumed sardines to increase their uric acid levels. Excess purine consumption with an impaired kidney also leads to gout and kidney stones, and so tracking these conditions daily is needed to ensure homeostatic kidney functioning.^[^ ^68^ ^]^ The UA sensor response changed by ≈7.5 µ*A*/cm^2^ and ≈11 µ*A*/cm^2^ in subjects 1 and 2, respectively, while the blood UA concentration changed by ≈0.7 − 1 *mg*/*dL* in both subjects. Moreover, the blood UA concentration peaked ≈120 and ≈75 min for subjects 1 and 2, respectively, after sardine consumption and its trend followed a slower decay rate (vs glucose). The variations in the peak timelines can be attributed to the interpersonal UA metabolism rate. Sweat and blood UA temporal trends correlated with each other with the peak lag time ranging ≈40–45 min. Such trends have been observed in sweat previously, which also reveals the slow metabolic rate of dietery purines and slow clearance rate of UA from blood to sweat in the body.^[^ ^58^, ^69^ ^]^ Elevated UA levels also contribute to increase in both MAP and HR. MAP increased by ≈12 mmHg with TEMPT and ≈8 mmHg with the cuff, ≈60 min before the blood UA peak in subject 1, while subject 2′s MAP increased by ≈8 mmHg with TEMPT and 6 mmHg with the cuff ≈20 min after the blood UA peak. Even HR increased by ≈15 bpm in subject 1 and correlated to the MAP trend. Such behavior is attributed to a) altered Renin‐Angiotensin System (RAS), where higher renin release in kidneys (from high UA) elevates the production of angiotensin II (a vasoconstirctor) in the lungs, eventually leaing to increased BP and b) elevated oxidative stress from reactive oxygen species generation.^[^ ^70^ ^]^ While the consistency of UA metabolism may vary in different subjects, the UA‐MAP profiles certainly provide unique insights into the complexity of purine metabolism and its potential impact on cardiovascular regulation. Hence, personalized management of UA related hypertension is crucial, which TEMPT can deliver.

Cortisol plays a key role in various physiological processes and its disruption due to chronic stress, disease, and aging has proven to have several implications on human health.^[^ ^71^ ^]^ CPT is traditionally used to elevate cortisol levels at rest since it triggers the pituitary glands to release the adrenocorticotropic hormone, which in turn stimulates the adrenal glands to relase cortisol in the bloodstream.^[^ ^72^ ^]^ CPT was conducted on two subjects following the testing timelines derived from Figure 3c,d (Figure 4c). Both subjects demonstrated peak sweat cortisol level after ≈20 min post CPT (with net changes ≈2 µ*A*/cm^2^ in subject 1 and ≈0.2 µ*A*/cm^2^ in subject 2) (Figure 4c). This timeline matches well with other sweat cortisol reports (peak timeline ≈10–20 min).^[^ ^42^, ^56^ ^]^ The touch cortisol sensor response was validated with osmotically extracted sweat (OES) using a competitive immunoassay. The corresponding immunoassay calibration is shown Figure S24 (Supporting Information).^[^ ^42^, ^73^ ^]^ OES was used since osmosis has been established to harvest sweat passively.^[^ ^73^, ^74^ ^]^ OES cortisol concentration changed by ≈50 and ≈90 nM in subjects 1 and 2, respectively, within 20 min after CPT. TEMPT and cuff MAP changed by ≈6 and ≈10 mm Hg, respectively in subject 1, while ≈4 and ≈8 mm Hg in subject 2. MAP increases due to elevated cortisol can occur from sustained adrenaline release in brain, altered RAS balance, or inhibited vasodilator release in blood.^[^ ^75^ ^]^ Thus, TEMPT MAP trends from CPT hold good correlation with the sweat cortisol trends. HR change was negligible in both subjects. Although, the direct correlation between cortisol and BP is an established metric in hypertensive individuals, TEMPT's capability to measure this in healthy individuals justifies its practical utiility for daily health tracking.

### 2.4 Extended Evaluation of TEMPT

**Figure 5:**
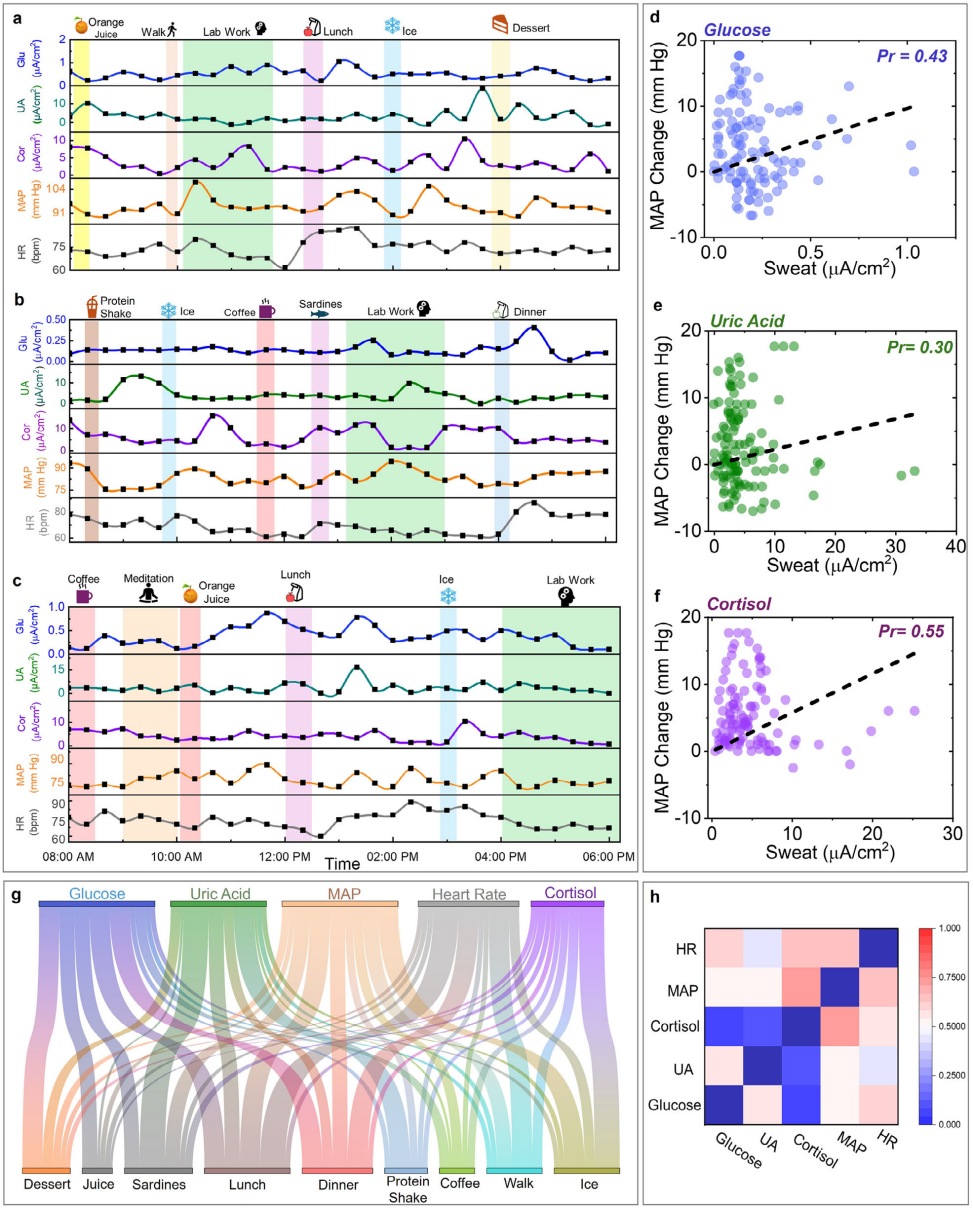
Extended study and statistical analysis of TEMPT a) Extended validation of TEMPT for 10 h under varying physiological activities throughout the day. Plot showing the temporal profile of all five markers with common stimuli such as drinking juice, meals, lab work, and dessert. CPT was added to elevate cortisol. b,c) New stimuli, such as protein shake, coffee, meditation, sardines, and dinner, were also added. d–f) Plots correlating the change in MAP with the change in metabolite levels, as obtained from all on‐body studies. Results show that cortisol has the highest effect on MAP during daily activities. Pr evaluation was conducted by fitting a line through the origin since we wanted to see to what extend change in MAP would directly correlate to the sweat metabolite levels. Glucose and UA have similar effects on the MAP change. n = 110 (glucose), n = 120 (UA), n = 110 (cortisol). g) Sankey diagram depicting the relative contribution of each stimulus on every biomarker, as measured by TEMPT from all on‐body studies. h) Heat map showing the normalized interdependence of all markers with each other. Information acquired from these plots elevates our understanding about the factors that majorly affect our daily health.

TEMPT was used to track the metabolic and vital sign profiles during daily activities. The goal of such tests was to explore the practical utility of TEMPT as a full scaled device to assist users with their daily health tracking. Multiple stimuli which are commonly experienced by users during work hours were chosen for such validation (**Figure** **5**a–c). In the first case study, the subject consumed orange juice in the morning, conducted regular lab work with mild walking, consumed lunch, did CPT, and then had dessert (Figure 5a). Sweat glucose response changed the highest with lunch (≈ 0.8 µ*A*/cm^2^), followed by dessert and juice. The minimal effect of juice is evident from the trends in Figure 4a. UA increased the highest from lunch (≈ 16 µ*A*/cm^2^) and it peaked after ≈2.5 h (surpassing the CPT test). Cortisol changed negligibly upon walking, but later increased from CPT. MAP and HR also showed an increasing trend from walking and lunch, possibly from the sodium and salt contents, which can suppress the dilation of blood vessels.^[^ ^76^ ^]^ However, CPT elevated MAP and not HR, which was also observed in Figure 4c. Next, additional effects of protein shake, black coffee, and sardines were evaluated in the second case study (Figure 5b). Glucose changed by ≈ 0.1 µ*A*/cm^2^ from sardines and maximum from dinner (≈ 0.4 µ*A*/cm^2^). UA increased significantly (by ≈10 µ*A*/cm^2^) with protein shake. This is evident as most commercial protein powders are derived from whey protein, which is a rich purine source. CPT had no effect on the UA levels. This also justifies the UA peak in the first case to be an outcome of lunch (despite appearing after CPT). Cortisol increased with coffee (≈ 7 µ*A*/cm^2^) as caffeine intake tends to stimulate the adrenocorticotropic hormone to secrete more cortisol.^[^ ^77^ ^]^ MAP increased majorly from CPT and sardines, while HR changed by a small amount from CPT and coffee. Finally, meditation was included in the third case study, where only MAP increased by ≈7–8 mm Hg (Figure 5c). Such changes can arise from the breathing style while meditating. In general, the initial cortisol levels in the morning were relatively higher due to the body's circadian rhythm in all three cases (Figure 5a–c).

The dependency of MAP change on the metabolic data was evaluated as the aim was to validate the hypothesis – change in sweat biomarker levels directly affects change in MAP. The net MAP change ranged from −7 to +15 mm Hg with sweat cortisol demonstrating the stronger correlation with MAP changes, followed by UA and glucose (Figure 5d–f). This validates that BP fluctuations are most sensitive to sweat cortisol changes during daily activities. Hence, the effect of CPT on MAP is greater than all other common stimuli (like meals, coffee, and juice), which is also observed in Figure 5a–c. These facts are further supported by the Sankey diagram shown in Figure 5g. This diagram summarizes the weighted contribution of each stimulus toward altering each vital and metabolic signature and supports understanding of the interplay of the signatures during such activities. Hence, in a decreasing order of effect, sweat glucose response increased mostly with meals, sardines, and juice. UA changed majorly with sardines, meals, and protein shake. MAP changed majorly with meals, mild walking, and CPT. HR increased primarily from meals, walk, and minimally from CPT. Sweat cortisol and MAP were affected mostly by CPT. Finally, the inter metabolite correlation was investigated (Figure 5h). The change of sweat glucose displayed a stronger correlation with UA than cortisol, with multiple stimuli throughout the day affecting glucose and UA more than cortisol. UA also did not show good correlation with cortisol due to similar reasons. All biomarkers had a greater effect on MAP than on HR (Figure S25, Supporting Information). Overall, such intriguing insights about the interplay of the metabolic and vital profiles hold considerable potential toward elaborating the key roles of diet, lifestyle, medication, and activity on the daily health profile of an individual.

## 3 Discussion

To fully understand a person's health, it is important to track both biochemical signals and vital signs, as all these parameters are interconnected and are dynamically influenced by each other. Our new touch‐based hybrid platform offers rapid, simple and reliable on‐site health and wellness assessment, eliminating the need for cumbersome lab testing and long sample‐to‐answer times. Such touch‐enabled hybrid system has been realized by overcoming the technological challenges of integrating optical detection of key physiological signals and electrochemical sweat metabolites measurements onto a single touch‐enabled platform. TEMPT makes this possible in a convenient and non‐invasive way by providing real‐time simultaneous estimation of crucial metabolic indicators (like glucose, UA, and cortisol from natural sweat) and vital signs (like MAP and HR) with just a simple finger touch. Our multi‐source real‐time data clearly demonstrate that this touch‐enabled self‐testing hybrid platform supports understanding of the interplay between biomarkers and vital signs during diverse daily activities.^[^ ^65^, ^66^ ^]^ The ergonomic design of TEMPT allows multimodal integration of physical and chemical sensors, making it suitable for convenient daily usage by all age groups. Our studies also show that TEMPT can distinctly highlight how different daily activities impact each biomarker and vital sign. These findings reveal that glucose, UA, MAP, and HR vary frequently throughout the day, with MAP being more closely linked to changes in sweat cortisol levels. Also, the sweat biomarkers of interest tend to affect MAP more than HR. Such capabilities make TEMPT a valuable tool for tracking metabolic and cardiovascular disorders. Lessons from diabetes have already proven that an ideal “*closed‐loop*” system (such as an artificial pancreas) cannot function based on only glucose and insulin levels.^[^ ^78^ ^]^ Hence, multiplexed data acquisition of several chemical and physical indicators is necessary for accurate health predictions and optimal interventions. Multiplexed data can be readily achieved with TEMPT in a user‐friendly way. The diagnostic platform can even be expanded to different key biomarkers and physical signals and customized to meet specific health and wellness monitoring needs (e.g., the management of diabetes, Parkinson or sepsis diseases). We believe that TEMPT can provide further unique information if tested on subjects with preexisting conditions and under the influence of stress and physical activity. Future large‐scale validation studies will further confirm the accuracy of the BP algorithm and would facilitate the integration and use of machine learning toward enhanced prediction of personalized health outcomes. Such approaches will empower users to take charge of their health and wellness through data‐driven insights, which could be beneficial for tracking wellness and fitness levels, managing chronic conditions, and making necessary lifestyle and dietary adjustments.

## 4 Experimental Section

### Materials and Apparatus

Glucose oxidase (GOx, from Aspergillus Niger, Type X‐S (EC 1.1.3.4)), bovine serum albumin (BSA), chitosan (medium molecular weight), nafion (perfluorinated resin solution), FeCl_3_, 6‐mercapto‐1‐hexanol (MCH), tris(2‐carboxyethyl) phosphine hydrochloride (TCEP), 2‐amino‐2‐(hydroxymethyl)‐1,3‐propanediol (Tris base), sodium chloride (NaCl), potassium chloride (KCl), magnesium chloride (MgCl_2_), hydrogen peroxide, glucose, cortisol, uric acid and l‐ascorbic acid were purchased from Sigma‐Aldrich. The cortisol aptamer probe (5′‐HO‐(CH_2_)_6_‐S‐S‐(CH_2_)_6_‐CTCTCGGGACGACGCCAGAA GTTTACGAGGATATGGTAACATAGTCGTCCC‐MB‐3′) synthesized by Integrated DNA Technologies (IDT) Inc. (Coralville, IA) was used as received. Phosphate buffered saline (1X PBS, pH 7.4) was acquired from Gibco. HPLC grade 2‐propanol and acetone, and hydrochloric acid (HCl) and nitric acid (HNO_3_) were purchased from Fisher Chemical. Gwent Carbon Graphite Mediated Paste (C2070424P2) was purchased from Sun Chemical and Ag/AgCl ink (E2414) was purchased from Ercon. Cortisol antibody (10‐C30D) and Cortisol‐3‐CMO‐HRP (65‐IC08) were acquired from Biosynth International (Louisville, KY). All electrochemical measurements were performed at room temperature using an EmStat3 Blue potentiostat (PalmSens, Netherlands) controlled by PSTrace software, version 5.9.

### TEMPT Fabrication

The TEMPT device fixture was fabricated using SLA 3D printing of 3 components: base, the PCB encapsulation and the sliding base for the MSA.

### Electronics Module

The fabrication of the TEMPT BP PCB consists of 2 major components: The BP PCB and Metabolic Sensor Array (MSA). The BP PCB was designed using Altium Designer and fabricated by an outsourcing manufacturer (PCBWay Inc., China) for assembly and fabrication. The board's dimensions are ≈3.67 cm x 6.73 cm. A 2 × 7 array of pins were left open for the soldering of the microcontroller (MCU) (Seeed Studio, XIAONRF52840, China). The MCU's USB‐C connection served as the charging, firmware, and data acquisition source. The MCU was reprogrammed to enable another I2C bus for the second. Two optical sensors (MAX30101, Maxim Integrated) were spatially separated by 5.5 cm. The MCU was programmed to enable another I2C bus for the second MAX30101 sensor. The BP PCB schematic and power consumption chart can be in Table S1 (Supporting Information).

### Calculation of Heart Rate (HR)

The filtering process deployed is explained earlier in Figure 2. Data collection was performed via Arduino IDE or PuTTTY serial logger. After the data acquisition process, a MATLAB script is executed to compute the PWV and output information regarding MAP, SBP, DBP, and HR. The HR was calculated using peaks detected from filtered photoplethysmogram signals. Raw PPG signals were first filtered using a two‐stage Butterworth filter. In the first stage, a 2nd‐order bandpass Butterworth filter was applied with cutoff frequencies set to 0.5 and 3.5 Hz to retain the primary cardiac components. A subsequent 3rd‐order low‐pass Butterworth filter with a 0.05 Hz cutoff frequency further smoothed the signals, reducing noise. Peak detection (in this case is the systolic peak) was performed on the filtered PPG signals using the *findpeaks* function with parameters for minimum peak height and minimum peak distance to identify significant peaks corresponding to heartbeats. For each segment, the time intervals between consecutive peaks were computed, yielding the peak‐to‐peak intervals, which represent the time between heartbeats in milliseconds. The average peak‐to‐peak interval for each PPG signal was then calculated by taking the mean value of these intervals and converting the result to seconds. The heart rate for each PPG signal was determined by dividing 60 by the mean interval (in seconds), yielding a heart rate value in beats per minute (BPM). This process was repeated for three data segments to obtain individual HR estimates from each segment of both PPG signals. Finally, the HR values for each signal across segments were combined and averaged to produce an overall HR measure. This approach allowed for an accurate HR calculation based on the average time between detected peaks, ensuring a robust measure across multiple data segments. A typical PPG obtained from the TEMPT device with annotated features can be seen in Figure 2a to better illustrate the features of interest in the HR and BP sensing schemes.

### PPG Pressure Study

An Imada force measurement tool was used to calibrate and monitor the pressure applied to the PPG sensors during testing (Figure S6, Supporting Information). The sensors were integrated into a custom setup where the calibrated force sensor was positioned directly beneath the PPG sensors to continuously measure the applied pressure. During testing, 3 different types of pressures (low, medium, high) were applied manually using the index and ring fingers, mimicking typical sensor contact within the TEMPT device (Figure S7, Supporting Information). The force sensor recorded the real‐time pressure that was plotted against PPG‐BP values. PPG, BP, and HR waveforms were collected under varying applied pressures to assess their quality and to evaluate the robustness of the extraction algorithms.

### MSA Chip Fabrication

The glucose and UA sensors rely on a two‐electrode system, while the cortisol sensor is a three‐electrode system. The disposable single‐use metal electrode arrays with Pt WE for glucose and Au WE for cortisol (12.5 mm^2^ area), a Au CE and a Ag/AgCl RE were designed in‐house as below. For this process, a 1.0‐mm‐thick PETG (glycol‐modified polyethylene terephthalate) sheet (Small Parts Inc.) was used as the substrate. A Cricut cutting machine was used to generate the electrode pattern design on protective laminated cover of the PETG substrate. Metal electrodes were obtained by sequential deposition of Cr (300 W for 6 min) and Au (100 W for 15 min) and Ag (100 W for 15 min) layers in direct current (DC) mode for Cr and Au and in radio frequency (RF) mode for Ag. The process deployed an Ar gas pressure of 2.4 mTorr, using a Denton Discovery 635 sputter system. The Ag layer on the WE and CE was removed by reacting 5 µL of 6 m HNO_3_ solution for 5 min, and the Ag surface of the RE was subsequently modified with 5 µL of FeCl_3_ for 2 min to obtain the Ag/AgCl‐based layer. The electrodes were cleaned before modifications by immersing them in solutions of isopropyl alcohol and water, each for 10 min.

### UA Sensor Chip Fabrication

The uric acid sensor is a two‐electrode system screen printed on a sheet of polyethylene terephthalate (PET). The two electrodes have Ag/AgCl current collectors that are initially screen printed on PET before screen printing the two different inks for the electrodes over the current collectors with sufficient overlap. The working electrode is made by screen printing Gwent graphite mediated ink, which is a carbon ink containing Prussian blue. The reference electrode is made by screen printing Ag/AgCl ink. After each ink layer is printed, the sensors are cured in the oven at 80 °C for 10 min.

### Drop Casting: Glucose

The working electrode on the glucose sensor was functionalized by successive drop casting of three layers in the order: 5 µL of glucose oxidase solution (40 mg mL^−1^), 1 µL of glutaraldehyde solution (1 wt.% in DI water), and 1 µL of chitosan solution (1wt.% in 0.1 m acetic acid). Each of the additions were followed by drying under a fan at room temperature before adding the next layer. All sensors used for testing were incubated for ≈4 °C overnight and used the following day.

### Uric Acid

The uric acid sensor was functionalized by drop casting on the working electrode, in order, 5 µL of uricase solution (15 mg mL^−1^), 1 µL of glutaraldehyde solution (1 wt.% in DI water), and 1 µL of chitosan solution (1wt.% in 0.1 m acetic acid). After each addition, the sensor was dried under a fan at room temperature. After drop casting, the sensors were kept at ≈4 °C overnight and used the following day.

### Cortisol

The fabrication of the cortisol sensor includes several steps. Before immobilizing the aptamer probe on the working electrode (WE3), thiol reduction was performed with 10 mm TCEP for 1 h at room temperature (dark environment) to allow covalent binding to the electrode surface. In detail, 2 µL of a 100 µm cortisol aptamer solution was mixed with 2 µL of 10 mm TCEP. Next, the solution was diluted with 50 mm Tris buffer containing 10 mm KCl, 100 mm NaCl, and 50 mm MgCl_2_, pH 8. Next, the cortisol aptamer probe solution was further diluted to 300 nM for further aptamer immobilization. Subsequently, the electrodes were incubated overnight at ≈4 °C. After immobilization of the aptamer, the electrodes were thoroughly washed with PBS and passivated with 15 µL of 3 mm MCH for 5 h at ≈25 °C. After MCH immobilization, the electrodes were washed with PBS and used in the following day.

### Hydrogel Fabrication

Porous PVA Hydrogel: A PVA (MW: ≈89000 Da) solution (0.1 g mL^−1^), KOH solution (0.2 g mL^−1^), and sucrose solution (1.04 g mL^−1^) were prepared. Next, all three components were mixed in the mass ratio 2.16:2.75:1 in a beaker. 2 mL of the mixed solution were placed in a petri dish (35 mm in diameter). Next 250 mg (10 wt.%) of Triton X‐100 was added to each petri dish and mixed gently. The gels were dried at 50 °C for 90 min, and then washed in DI water until a neutral pH was reached. Before tests, the hydrogel was equilibrated with 1X PBS for 10 min.

### On‐body Testing Protocol: Glucose

All subjects fasted overnight before testing. Prior to testing, subjects’ fingertips were cleaned with alcohol wipes. Blood glucose levels were measured every 15 min using the “Accu‐Check Guide Me (Roche, Switzerland)” Monitor through finger pricking. Amperometric glucose detection was performed every 15 min using an EmStat Blue 3 potentiostat (PalmSensBV, Netherlands). For each sweat measurement the subject touched the sensor for 2 min. Next, the sensor incubated for 4 min, while covered, before taking measurements. After two data points were collected, subjects consumed carbohydrates (via fruit juice). Measurements of blood and sweat glucose continued to be taken every 10 min for 90 min after the carbohydrate intake. Blood pressure measurements were also made at 10‐min intervals using a cuff and optical system.

### Uric Acid

Prior to testing, subjects’ fingertips were cleaned with alcohol wipes. Blood uric acid levels were measured every 20 min using the “UA Sure II Blood Uric Acid Monitoring System (Atwood, CA)”. Amperometric uric acid detection was performed every 20 min using an EmStat Blue 3 potentiostat. The subject touched the sensor for 2 min, followed by a 2‐min incubation period before amperometric measurements were carried out. Once two data points were collected, uric acid was consumed (via sardines), and measurements of blood and sweat uric acid continued to be taken every 20 min for 200 min after the uric acid intake. Blood pressure measurements were also performed every 20 min with a cuff and optical system.

### Cortisol

Prior to testing, subjects’ fingertips were cleaned with alcohol wipes. For the validation, osmotically extracted sweat cortisol levels were measured every 10 min using the cortisol immunoassay. Sweat cortisol acid levels were measured every 20 min. The subject touched the sensor for 2 min, followed by a 6‐min incubation period before SWV measurements were carried out. Once two data points were collected, cortisol was stimulated via CPT, and measurements of OES and sweat cortisol continued to be taken every 10 min for 60 min after CPT. Blood pressure measurements were also taken every 10 min with a cuff and optical system.

### Extended Study Protocol

All on‐body trials were conducted based on the approved IRB (#130003, UCSD). Written consent was acquired from all participants before the test. Verification of the extended operational validity of TEMPT system was tested under daily activities and the intake of multiple meals. Subjects underwent overnight fasting to prepare for the test. Prior to beginning the test, subjects washed their hands with soap and water and their fingertips were cleaned with alcohol wipes. Measurements of sweat cortisol, uric acid, and glucose were performed every 20 min using an EmStat Blue 3 potentiostat or PalmSense4. Glucose and uric acid sensors were pressed for 2 min followed by a 4‐min incubation for glucose and 2‐min incubation for uric acid. Blood pressure measurements were also made every 20 min. Cortisol sensors underwent 2 min of contact and 6 min of incubation. One to two data points were collected before any stimuli were consumed to establish a fasting baseline. Blood glucose and uric acid levels were taken whenever a spike in glucose or uric acid was anticipated. For the ice test in the extended studies, the subject submerged their right hand in a bucket of ice and water for 5 min. The finger that was used in the electrochemical sensing was covered in plastic wrap. The food and drinks that were consumed are specified in the figures corresponding to each study.

## Conflict of Interest

The authors declare no conflict of interest.

## Author Contributions

O.D., T.S., and P.N. contributed equally to this work. T.S., O.D, P.N, and J.W conceived the project idea. O.D., T.S., P.N., and M.I.K. designed the prototype. O.D., T.S., P.N., M.I.K., H.F., S.E., C.M., Y.X., H.T., R.P., B.A., S.M‐F., O.W., C.W., A.Y.C., C.C., J.S., S.D. performed experiments. T.S., O.D., and P.N., designed the human subject studies. O.D, T.S., P.N, and J.W analyzed the data. T.S., O.D. and J.W. wrote the paper and designed the figures. J.W. supervised the project. All authors discussed the results and commented on the manuscript.

## Supporting information



Supporting Information

Supplemental Video 1

Supplemental Video 2

## Data Availability

The data that support the findings of this study are available from the corresponding author upon reasonable request.
