# Supplementary material for: A Touch Enabled Hemodynamic and Metabolic Monitor
Source: Adv Sci (Weinh). 2025 Apr 17;12(26):2502138. doi: 10.1002/advs.202502138 (PMC12245003; doi:10.1002/advs.202502138)
Supplement: Supplementary file 1 — Supporting Information [file ADVS-12-2502138-s001.pdf]

ADVANCED SCIENCE
Open Access

Supporting Information

for *Adv. Sci.*, DOI 10.1002/advs.202502138

A Touch Enabled Hemodynamic and Metabolic Monitor

*Omeed Djassemi, Tamoghna Saha, Ponnusamy Nandhakumar, Muhammad Inam Khan, Hannah Fishman, Sara Earney, Chochanon Moonla, Yuchen Xu, Henry Thai, Sofia Morales-Fermin, Gyeongho Kim, Rhea Park, Beya Acot, Oscar Wu, Cannon Wurster, An-Yi Chang, Christopher Cheung, Julia Silberman, Shichao Ding and Joseph Wang**

Supplementary Information

A Touch Enabled Hemodynamic and Metabolic Monitor

Omeed Djassemi[ab$], Tamoghna Saha[a$], Ponnusamy Nandhakumar[a$], Muhammad Inam Khan[a], Hannah Fishman[a], Sara Earney[a], Chochanon Moonla[a], Yuchen Xu[b], Henry Thai[c], Sofia Morales-Fermin[a], Gyeongho Kim[a], Rhea Park[a], Beya Acot[a], Oscar Wu[d], Cannon Wurster[d], An-Yi Chang[a], Christopher Cheung[a], Julia Silberman[a], Shichao Ding[a], and Joseph Wang[a*]

[a] Aiiso Yufeng Li Family Department of Chemical and Nanoengineering, University of California San Diego, La Jolla, CA 92093, USA

[b] Shu Chien-Gene Lay Department of Bioengineering, University of California San Diego, La Jolla, CA 92093, USA

[c] Department of Mechanical and Aerospace Engineering, University of California San Diego, La Jolla, CA 92093, USA

[d] Department of Electrical and Computer Engineering, University of California San Diego, La Jolla, CA 92092, USA

[$] Equal Contribution

[*] Corresponding Author: josephwang@ucsd.edu

Table of Contents

Supplementary Figure 1: Components of TEMPT platform and circuit schematic of TEMPT BP/HR PCB.

Table S1: Power consumption metrics of BP sensor.

Supplementary Video 1: 360-degree view of TEMPT.

Supplementary Video 2: Simultaneous capturing of vital and metabolic signatures from TEMPT.

Supplementary Figure 2: MSA fabrication steps.

Supplementary Figure 3: Various LED PPG Data.

Supplementary Figure 4: Description of the PPG waveform from the TEMPT system.

Supplementary Figure 5: PPG from TEMPT PCB.

Supplementary Text 1

Table S2: Comparison of our BP metrics with literature.

Supplementary Figure 6: Calculating the PWV at various inter-finger distances.

Supplementary Text 2

Supplementary Figure 7: Pressure sensor calibration.

Supplementary Figure 8: Effect of pressure on the BP study.

Supplementary Figure 9: PPG Interference studies.

Supplementary Figure 10: PPG waveforms under varying skin tones.

Supplementary Figure 11: PPG waveform, MAP, and HR trends during exercise.

Supplementary Figure 12: Reusability of the cortisol aptamer sensor.

Supplementary Figure 13: In-vitro studies of the glucose sensor.

Supplementary Figure 14: In-vitro studies of the uric acid sensor.

Supplementary Figure 15: In-vitro studies of the cortisol sensor

Table S3: Comparison of uric acid sensor with literature.

Table S4: Comparison of glucose sensor with literature.

Table S5: Comparison of cortisol sensor with literature.

Supplementary Figure 16: Scanning electron microscopy (SEM) of hydrogels.

Supplementary Figure 17: Estimation of the hydrogel thickness.

Supplementary Figure 18: MB SWV measurements with and without the gel.

Supplementary Figure 19: In-vivo studies of the cortisol sensor

Supplementary Figure 20: Pressure analysis of the metabolic touch sensor.

Supplementary Figure 21: Effect of touch pressure on the glucose sensor response.

Supplementary Figure 22: Effect of touch pressure on the UA sensor response.

Supplementary Figure 23: Effect of touch pressure on the amperometric response.

Supplementary Text 3

Supplementary Figure 24: Cortisol immunosensor calibration.

Supplementary Figure 25: Effect of sweat biomarkers on heart rate.

A)

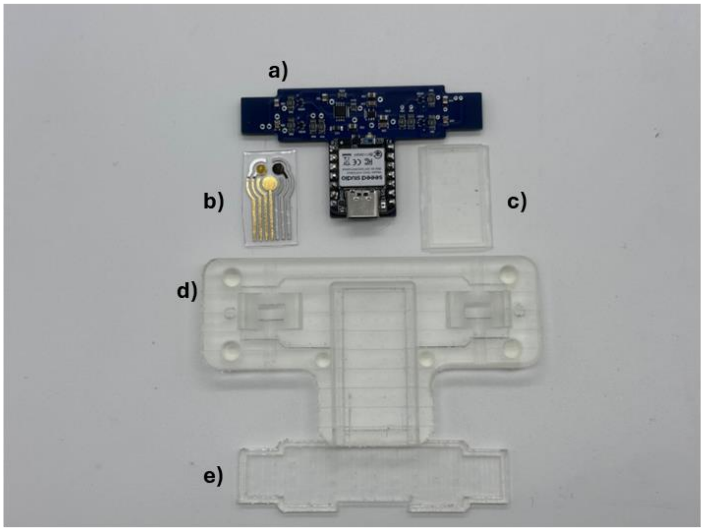

B)

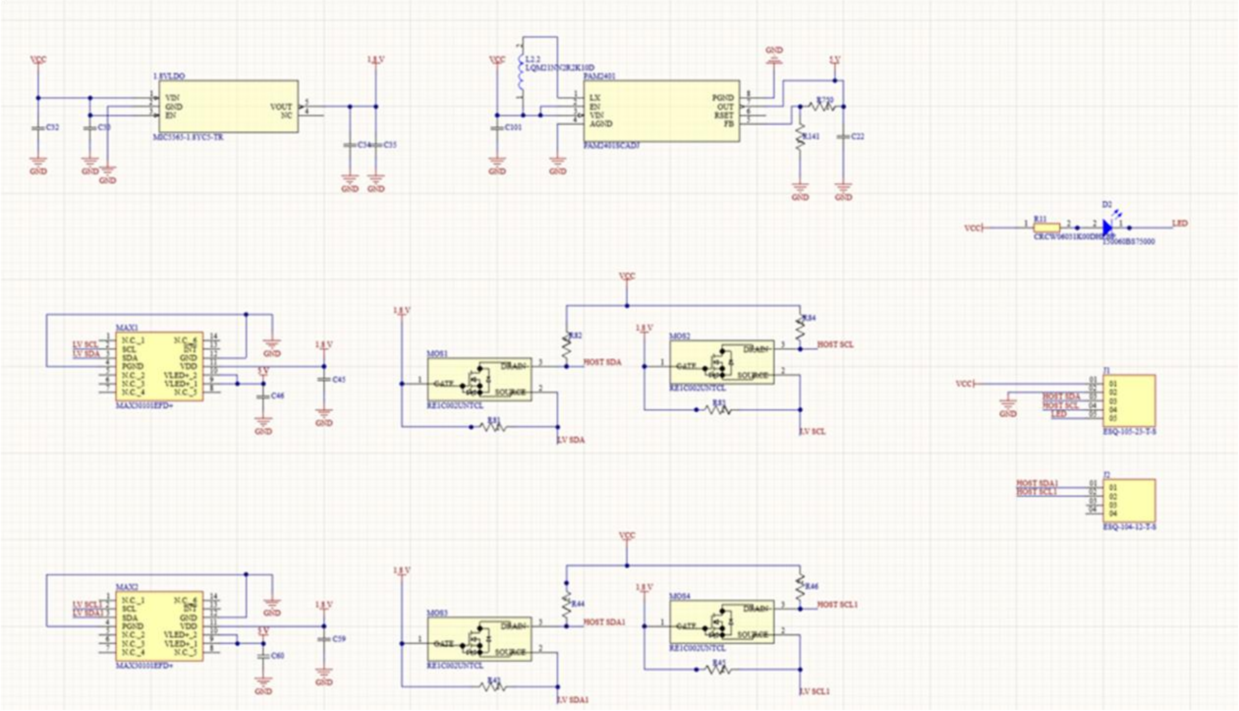

Fig. S1. Components of TEMPT platform and circuit schematic of BP/HR PCB. A) a) BP/HR PCB and BLE SoC. b) Glucose, Uric Acid, Cortisol sensor array. c) sensor holder. d) PCB and electrode holder. e) Back cover for PCB. B) Circuit schematic of the TEMPT BP/HR PCB.

LED	Current Consumption	Power Consumption (VCC = 5V)	Power Consumption (Vcc = 3.3V)
IR Sensors ON	13 mA	65 mW	43 mW

Table S1: Power consumption metrics of BP sensor.

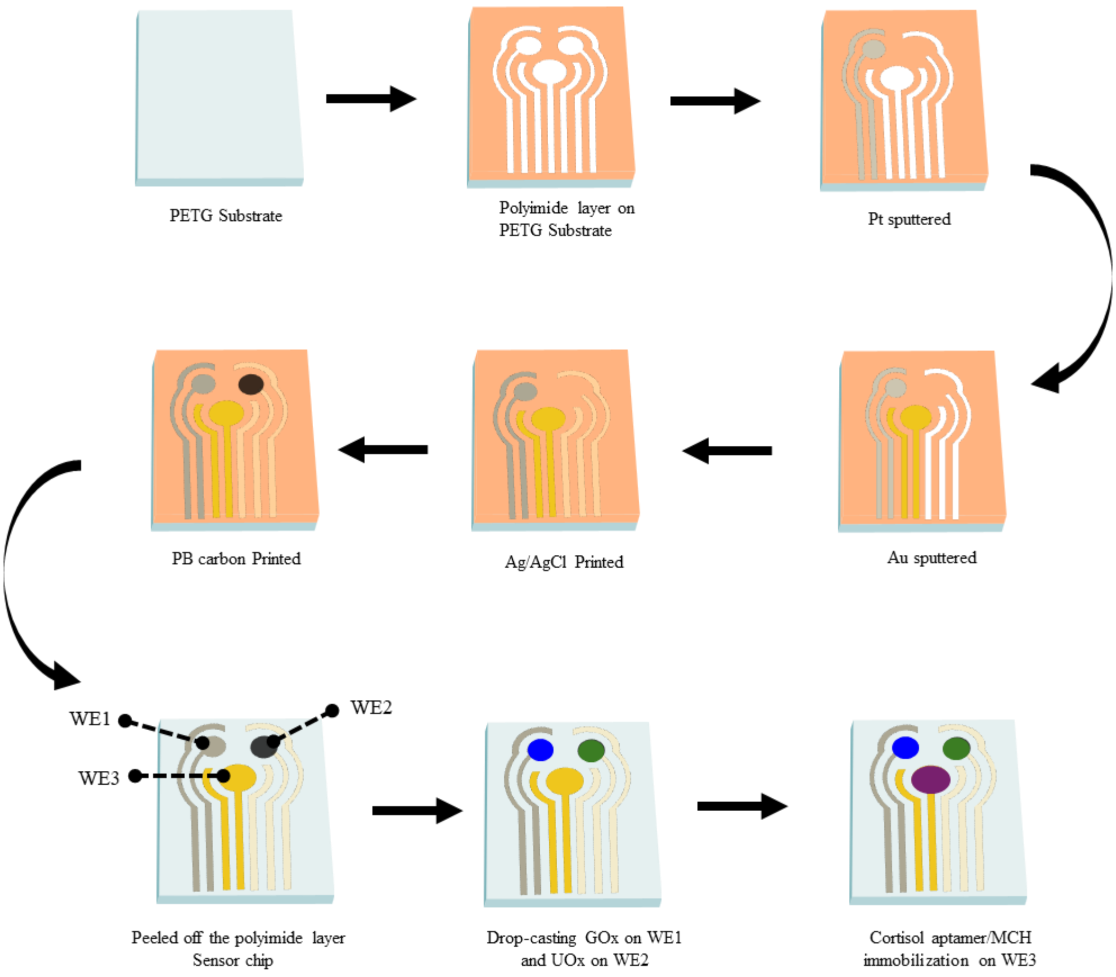

Fig. S2. MSA fabrication steps. Schematic showing the fabrication steps of the metabolic sensor array for glucose, uric acid, and cortisol. Blue – Glucose electrode, Green – UA electrode and Purple – Cortisol electrode.

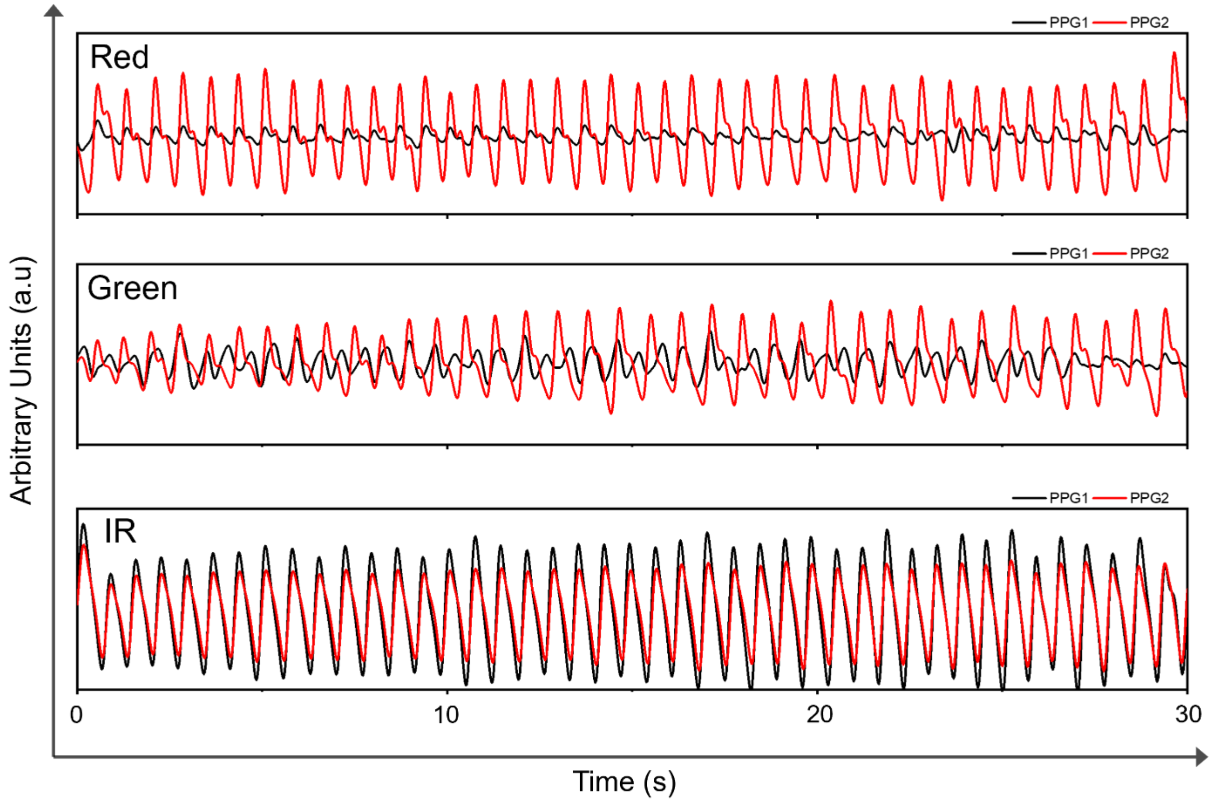

Fig. S3. Various LED PPG Data. 30 seconds of data acquisition for three different wavelengths of light. Infrared (IR) data demonstrating the highest prominence amongst the three light sources contained in the MAX30101.

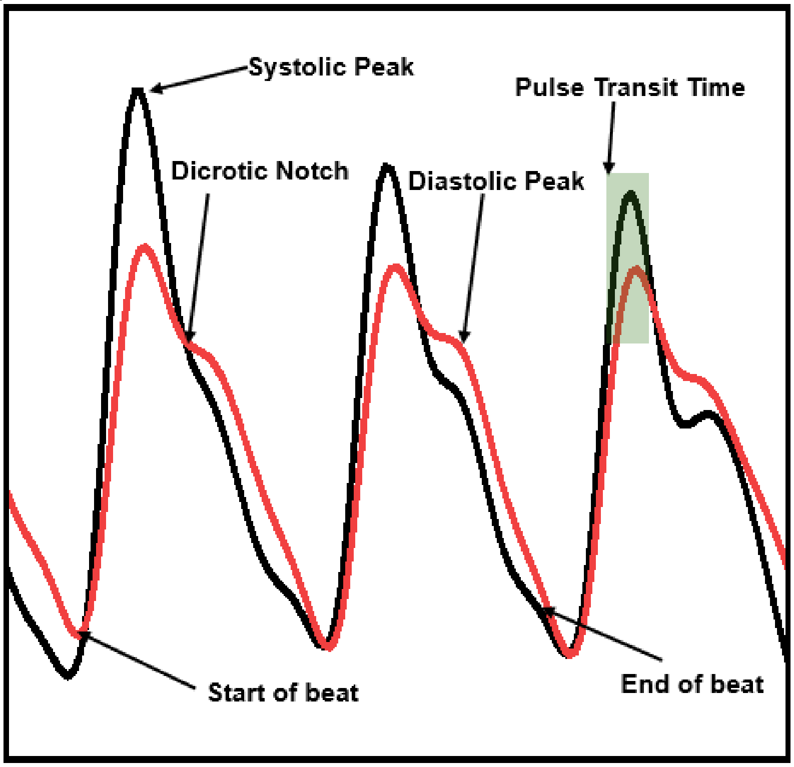

Fig. S4. Description of the PPG Waveform from the TEMPT system. An annotated PPG waveform obtained from the TEMPT platform. The PTT is used for BP analysis.

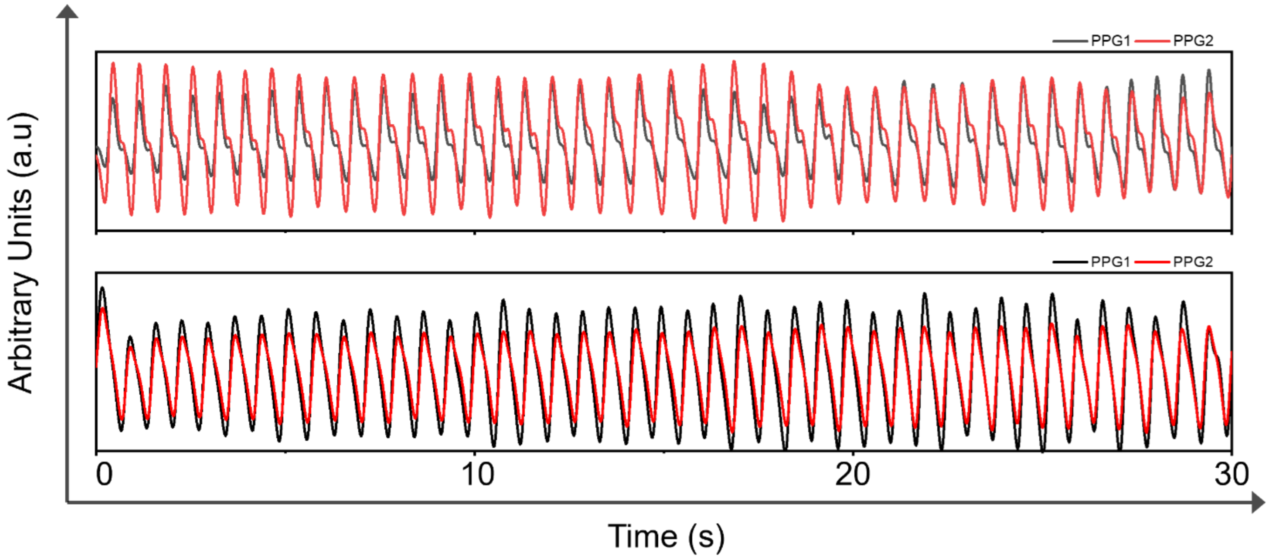

Fig. S5. PPG from TEMPT PCB. Comparison of PPG waveforms between the TEMPT PCB (top) and MAX30101 breakout board (bottom). Similar waveform trends justify the accuracy of TEMPT PCB.

Supplementary Text 1: Byfield et. al has previously demonstrated the approach of using PWV to estimate BP[1]. This supplementary note will touch upon the methods used for the genesis of the SBP and DBP second order polynomials used in this work and also discuss other key metrics. The same group correlated the PWV to SBP and DBP by taking 10 successive 12-second measurements and taking the average PWV and correlating it to the average BP Cuff metrics. This was repeated for 26 subjects to obtain a mathematical relationship between PWV and SBP/DBP. The equations resulted in second order polynomials described below:

1) $SBP = -1.28(PWV)^2 + 16.03(PWV) + 97.48$
2) $DBP = -0.61(PWV)^2 + 8.56(PWV) + 61.85$

It is important to note the range of blood pressures that are covered by these two equations, specifically calculating the maxima and minima of each equation to better assist in the screening of subjects that will fit into the algorithms outlined. The SBP algorithm gives a maximum value of 148 mmHg while the DBP maxima is 92 mmHg. These constraints were taken into consideration when choosing the stimuli across the work, with emphasis on the exclusion of exercise. Additionally, there are no cuff SBP values in this work that exceed these upper bounds of the algorithm.

Further optimization of the sampling frequency and distance between the two sensors can be tuned to expand the dual-PPG system beyond what has been shown previously on the fingers. The algorithms deployed in this work are only functional with a sampling frequency of 50 Hz. Tuning the sampling frequency for a user's needs may require the development of new BP algorithms and has the potential to give a more accurate BP prediction while expanding the linear range of the algorithm. Because we are calculating the PWV by using the equation:

3) $PWV = \frac{L}{\Delta t}$

The length can be adjusted to give more modular PPG-based systems across various epidermal locations.

1. Byfield, R., Miller, M., Miles, J., Guidoboni, G. & Lin, J. Towards Robust Blood Pressure Estimation From Pulse Wave Velocity Measured by Photoplethysmography Sensors. *IEEE Sens. J.* **22**, 2475–2483 (2022).

Table S2: Comparison of our BP metrics with literature

Reference	Calibration Free	ML Statistics	SBP/DBP error	Power
This work	Yes	No	5.35 +/- 1.08	43 mW
1	Yes	Yes	4.84 +/- 4.16	N/A
2	Yes	No	7.1 +/- 8.5	N/A
3	No	No	N/A	0.2 mW/cm^2
4	Yes	No	N/A	N/A

1. Matthews, Jared, et al. "Cloud-Integrated Smart Nanomembrane Wearables for Remote Wireless Continuous Health Monitoring of Postpartum Women." *Advanced Science* 11.13 (2024): 2307609.
2. Xuan, Yinan, et al. "Ultra-low-cost mechanical smartphone attachment for no-calibration blood pressure measurement." *Scientific reports* 13.1 (2023): 8105.
3. Chung, Ha Uk, et al. "Binodal, wireless epidermal electronic systems with in-sensor analytics for neonatal intensive care." Science 363.6430 (2019): eaau0780.
4. Franklin, Daniel, et al. "Synchronized wearables for the detection of haemodynamic states via electrocardiography and multispectral photoplethysmography." *Nature biomedical engineering* 7.10 (2023): 1229-1241.

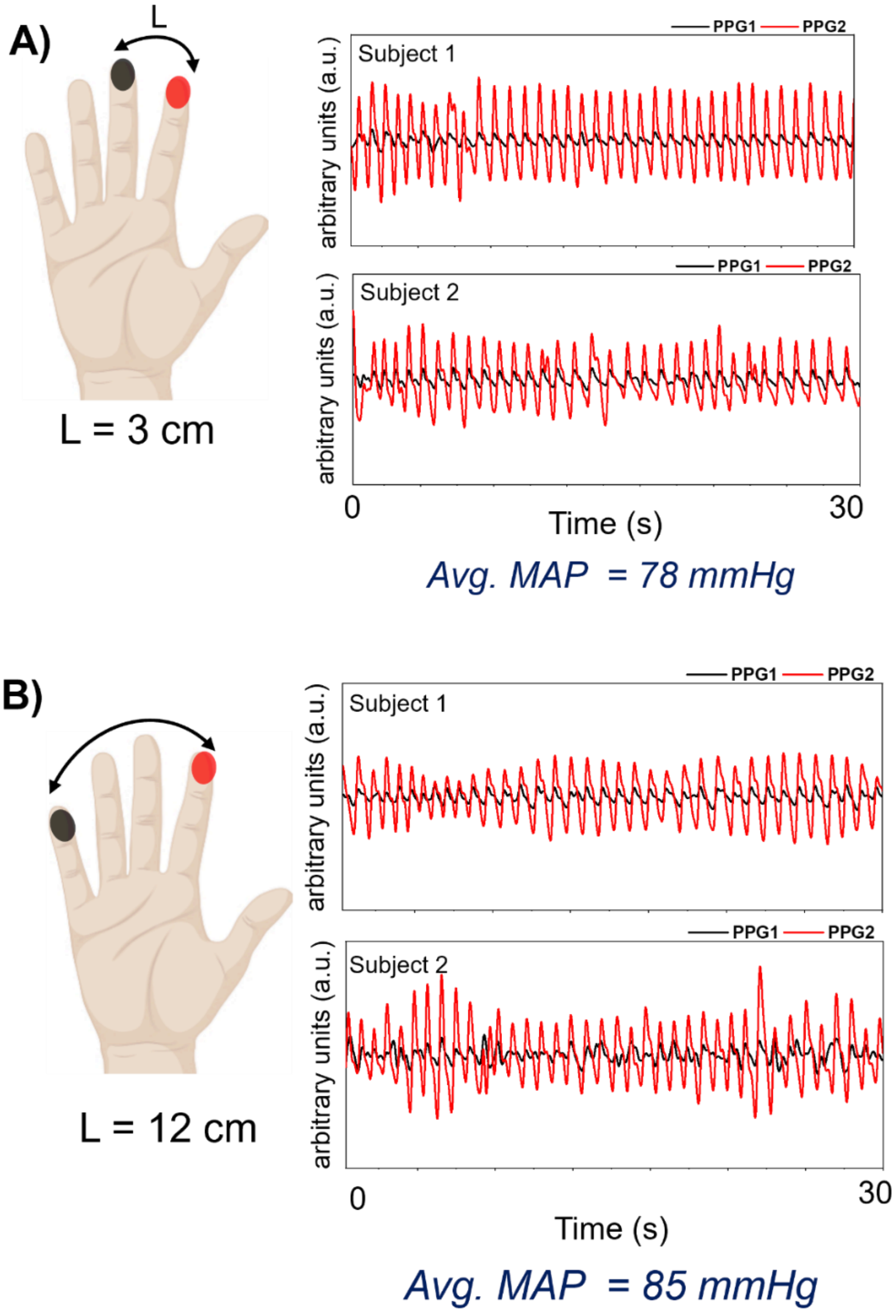

Fig. S6. Calculating the PWV at various inter-finger distances. A) MAP analysis of 2 subjects using the middle finger and index finger. The distance between the index and middle finger is 3 cm. b) MAP analysis of 2 subjects using the pinky and index finger. The distance between the two locations for this measurement was 12 cm. Results show that both PPG waveforms do not follow a similar trend, which proves that L being too close or too far are not good for reliable PPG acquisition.

Supplementary Text 2: The TEMPT system does not include any pressure sensing element. However, pressure validation is necessary with respect to the integrity of the PPG waveform. PPG sensors detect variations in blood flow and volume by emitting light (infrared or red) and measure the amount of light absorbed or reflected by blood. Excessive pressure can compress the blood vessels, reduce blood flow and result in weaker signals. On the other hand, too little pressure may lead to inconsistent skin contact, causing noise and signal loss. Hence, applying relative consistent pressure ensures the sensors to remain in stable contact with the skin and minimizes baseline fluctuations. Without stable contact, motion artifacts and signal variations may compromise the quality of the data.

It is also important to note that BP estimation tactics in this work are time-series based, which offers flexibility in maintaining consistent pressure to a PPG sensor, as they use continuous data over time to derive BP estimates. Time-series-based algorithms often employ advanced filtering and smoothing techniques to reduce noise and artifacts caused by inconsistent pressure. While optimal pressure ensures high-quality PPG signals, time-series methods can compensate for brief fluctuations or signal distortions by focusing on long-term trends and minimizing the impact of short-term irregularities. Instead of relying on a single point measurement, we take 30 second measurements and calculate BP values every 10 seconds to bolster the time-series algorithms and aggregate data over time. This continuous data collection creates redundancy, allowing the algorithm to average out variations caused by inconsistent pressure. As a result, transient pressure changes may have a minimal effect on the overall BP estimation. To further validate these comments, we conducted several experiments to show the impact of pressure on BP measurements, and most notably signify the hypothesis that subjects across the general population can be trained to apply the same amount of pressure on average for time-series based approaches for BP prediction. The results of the study with reported MAP percent error can be found in **Fig S8.**

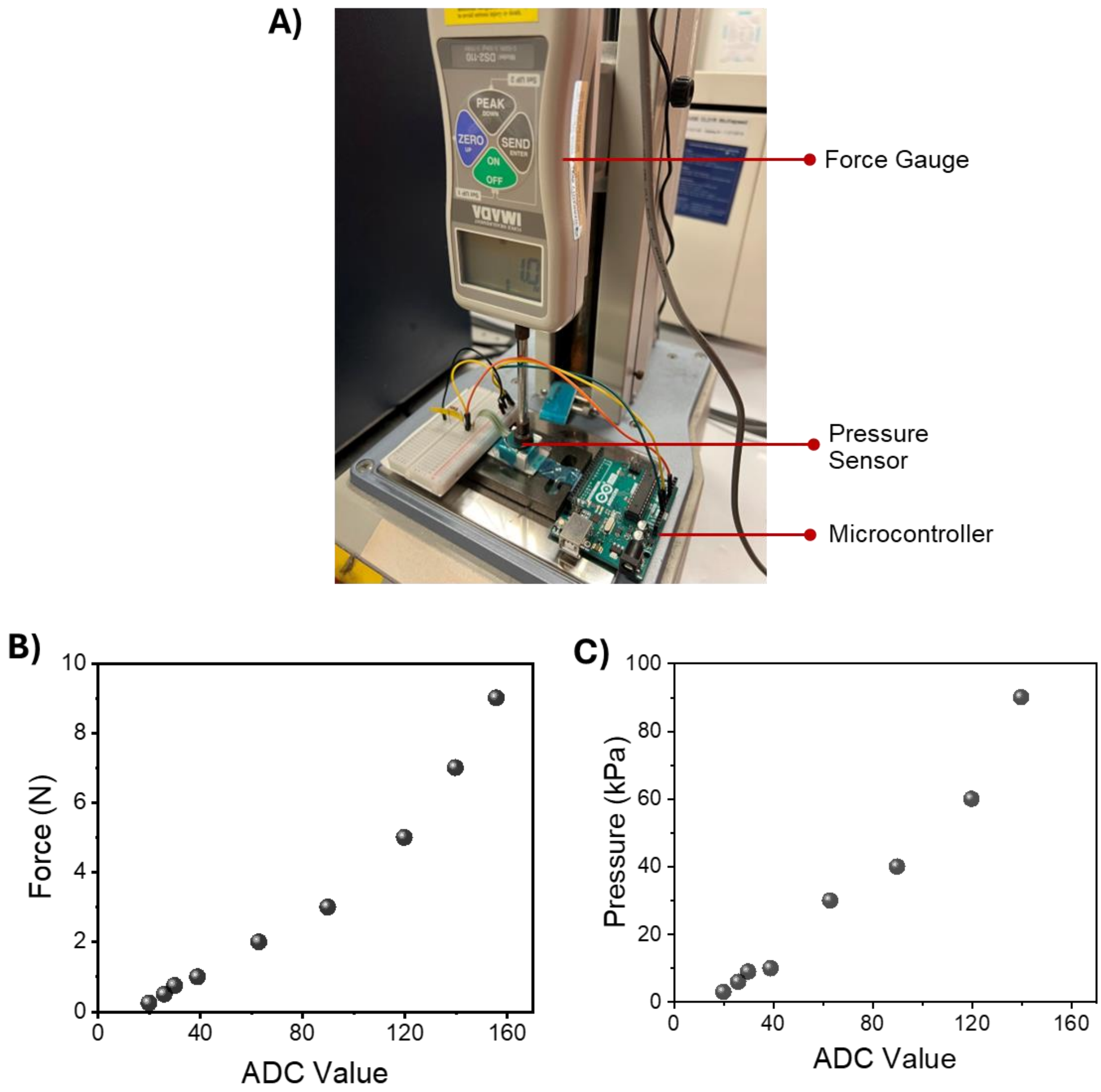

Fig. S7. Pressure sensor calibration. A) A pressure sensor calibration apparatus consisting of a force gauge, pressure sensor, and a microcontroller for data acquisition. B) Force calibration plot. Forces of 0.25 N, 0.5 N, 0.75N, 1 N, 3N, 5N, 7N, 9N were collected and plotted against an analog result. C) Pressure calibration plot. Force values were converted to pressure units by dividing the surface area of the pressure gauge of 0.785 cm^2.

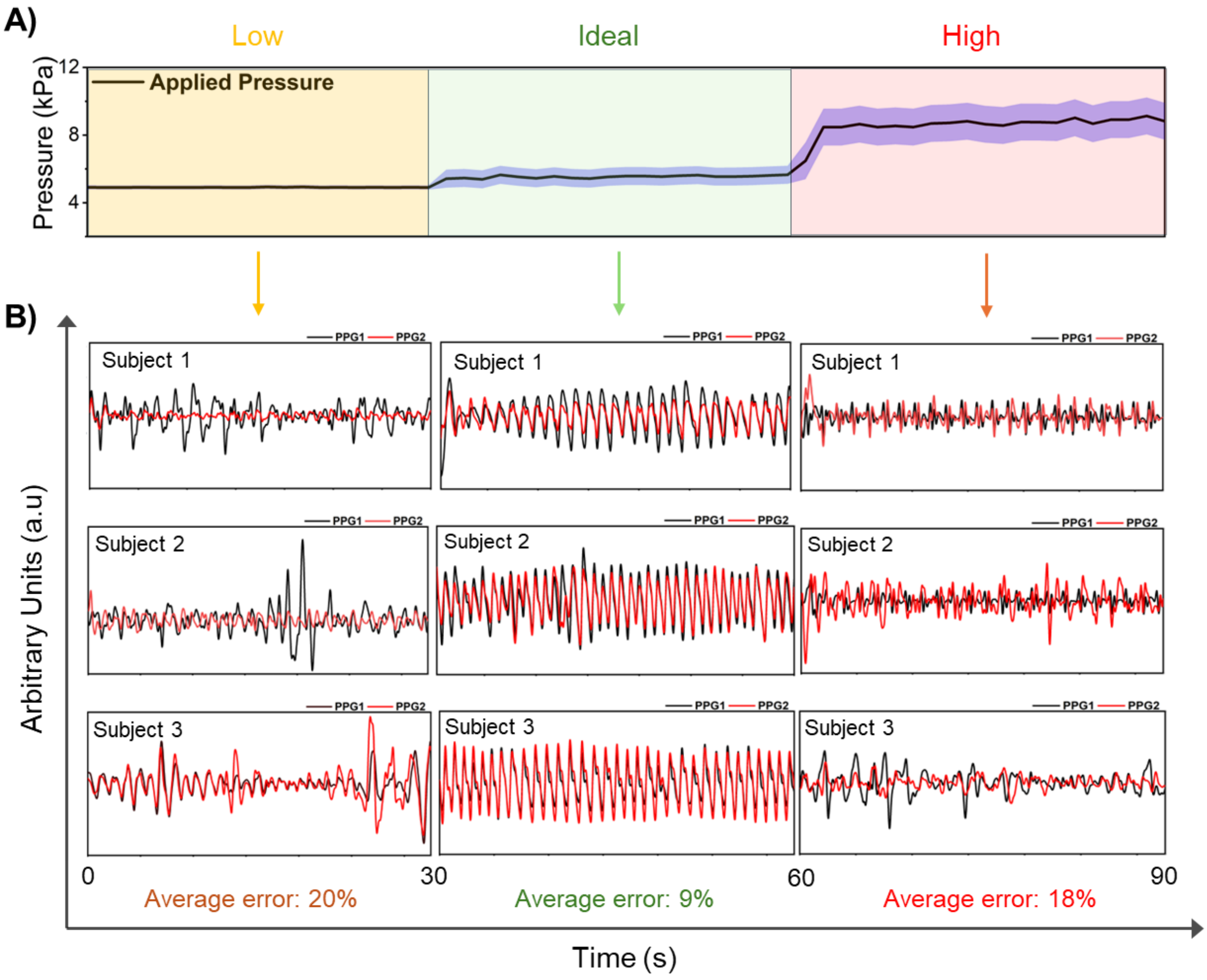

Fig. S8. Effect of pressure on the BP study. A) PPG waveforms from three subjects were evaluated with a pressure sensor integrated underneath the TEMPT system. The ideal pressure applied to the sensors was found to be 5.7± 0.08 kPa. The blue shaded region denotes the SD region. B) Both PPG waveforms show good correlation only in the ideal pressure zone. The average % error of the MAP was calculated with respect to the cuff MAP measurement.

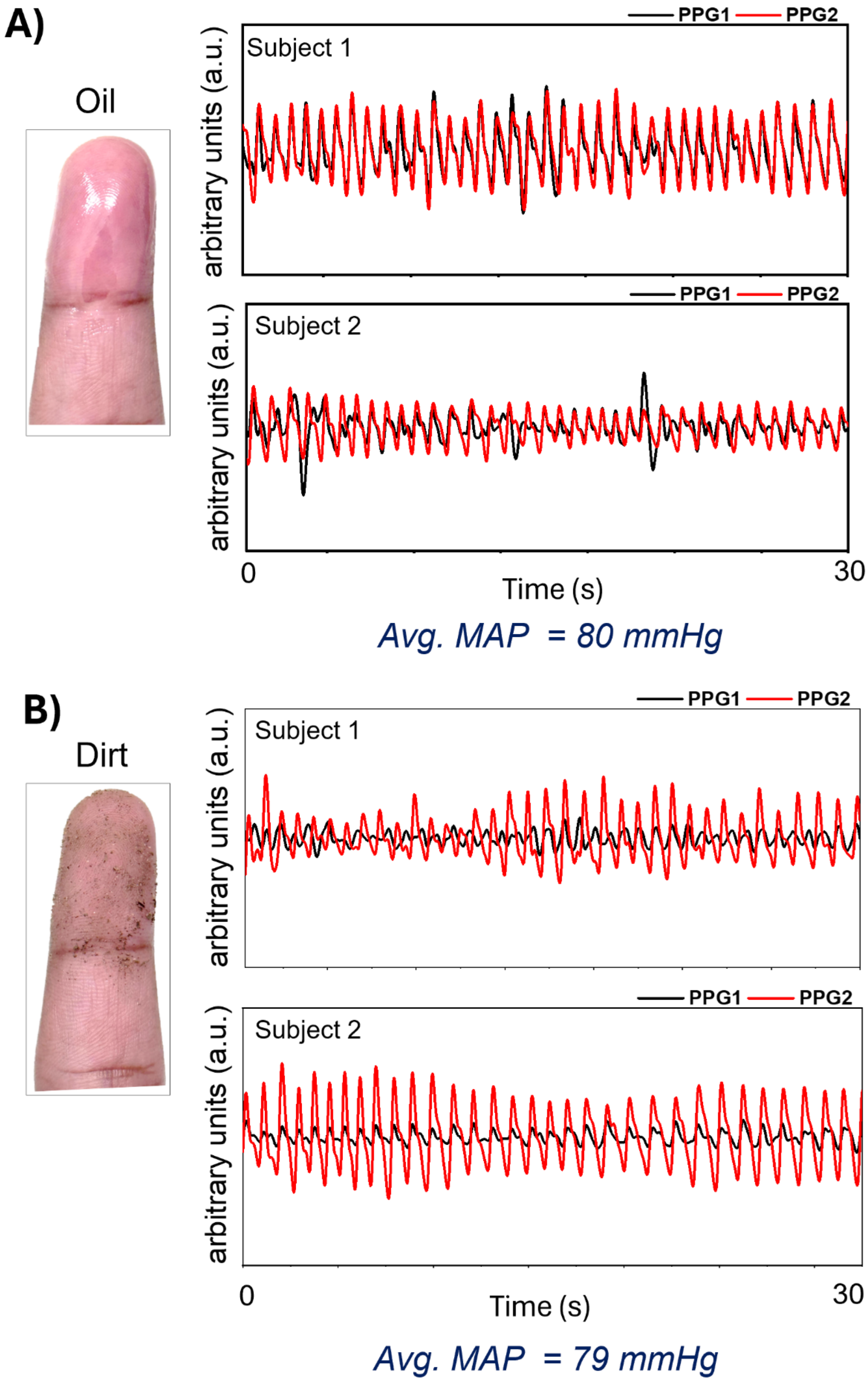

Fig. S9. PPG Interference studies. A) MAP analysis of 2 subjects using oil as an interferent to PPG signal acquisition. Average MAP of 80 mmHg falls within a healthy

range and can be deemed acceptable across two subjects. B) MAP analysis of 2 subjects using dirt as an interference to PPG signal acquisition. Similar to the oil study, subjects displayed an average MAP of 79 mmHg, signifying extraneous substances, like dirt and oil, do not affect the IR light penetration to the capillary bed for finding the PTT. However, measuring under such a situation is not recommended as both PPG waveforms do not align well with each other.

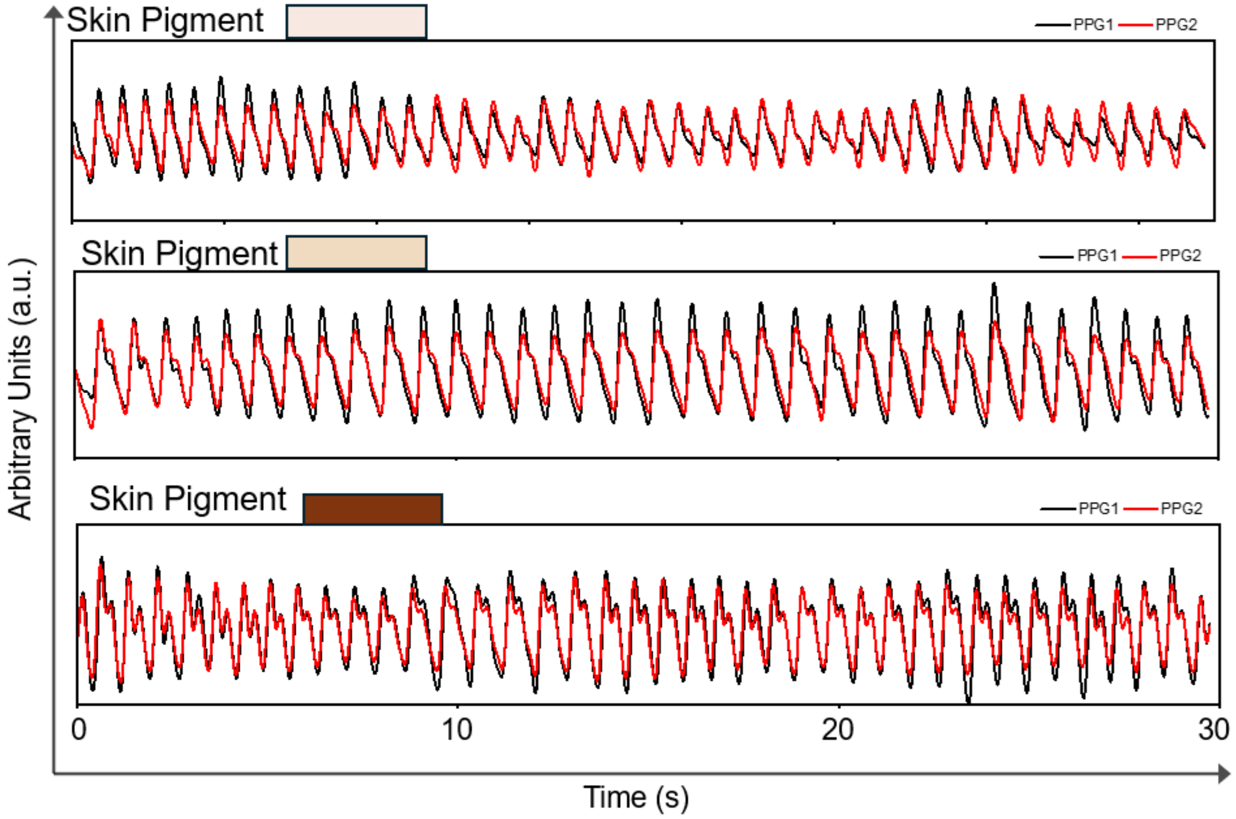

Fig. S10. PPG waveforms from varying skin tones. 30 seconds of data IR PPG acquisition for three different skin tones shows insignificant difference among different skin tones.

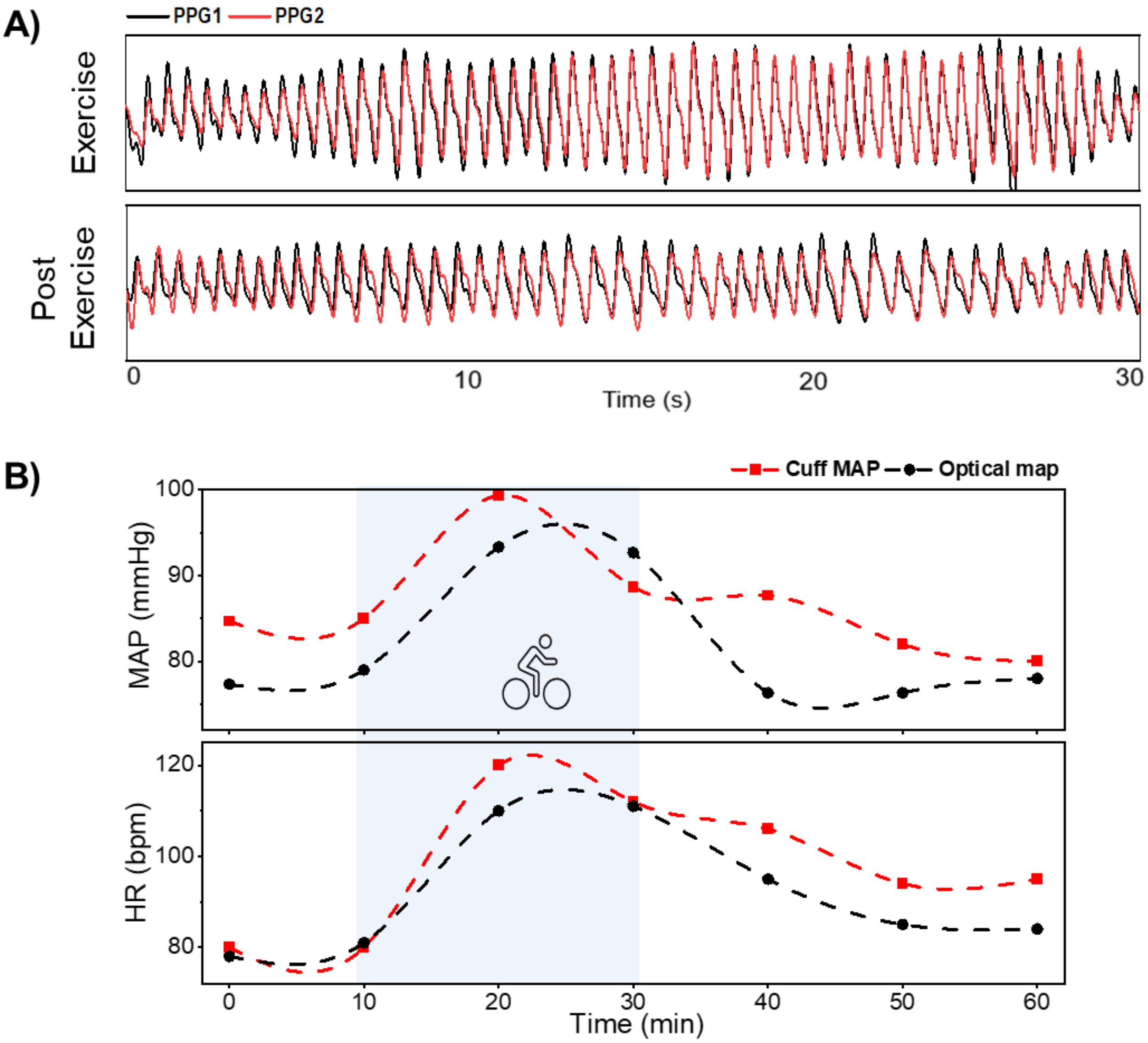

Fig. S11: PPG waveform, MAP, and HR trends during exercise. 20 mins of exercise shows similar PPG trends, but with a faster increasing rate of MAP. HR increased significantly (vs. rest).

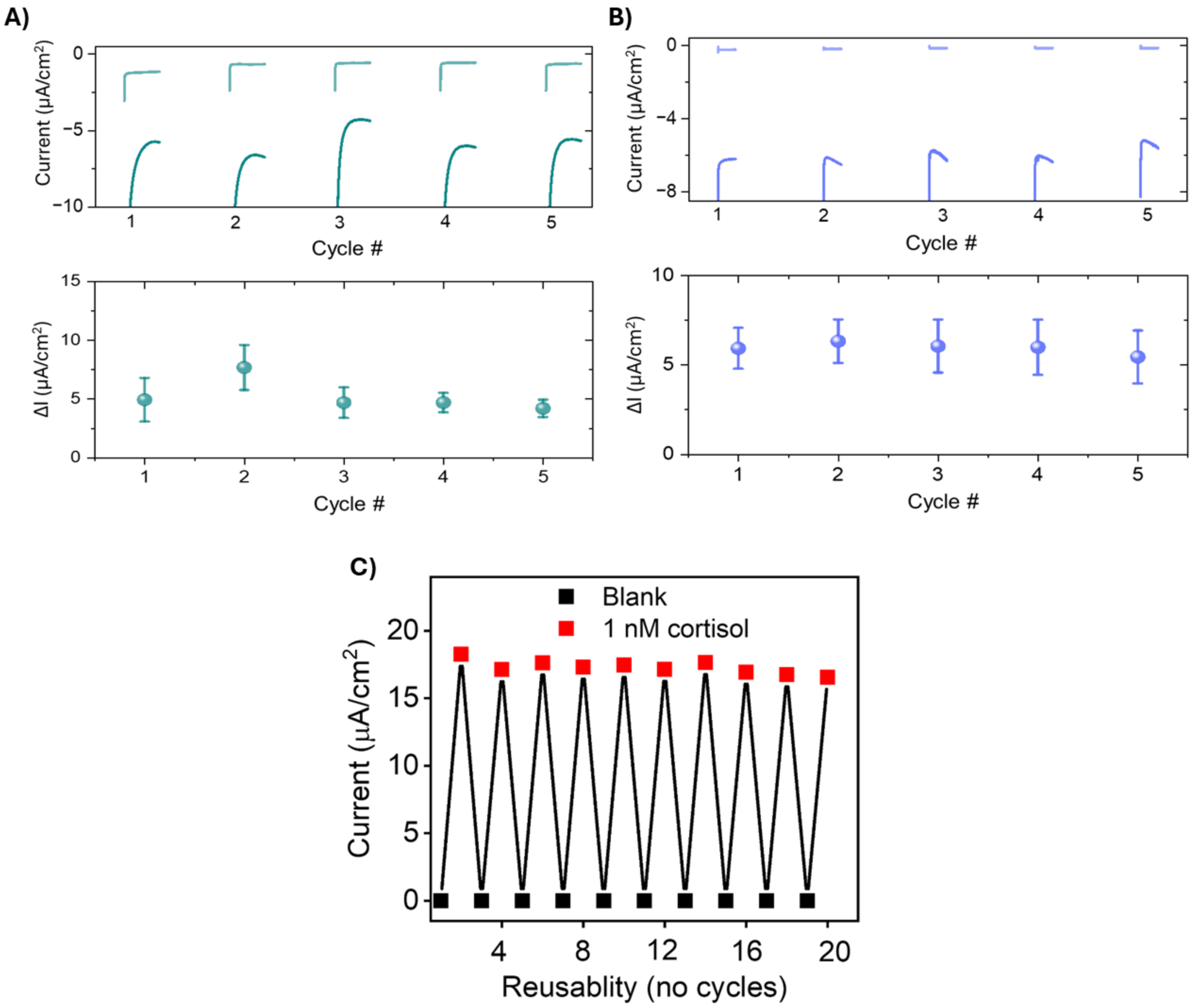

Fig. S12. Reusability metrics of all sensors. Plot showing the reversibility of A) uric acid and B) glucose sensor up to 5 cycles without washing the sensor surface. The spiked concentration was 200 µM in 50 µL's 0.1M PBS buffer. Light line: blank response, dark line: signal. We believe the reversibility limit of these sensors under real-conditions will be even longer as they will be operating with lower sweat volumes. (C) Current changes obtained from the in-vitro SWV response to 1 nM cortisol detection using same cortisol sensor. Results justify aptamer and enzymatic sensor stability and reversibility for long-term monitoring.

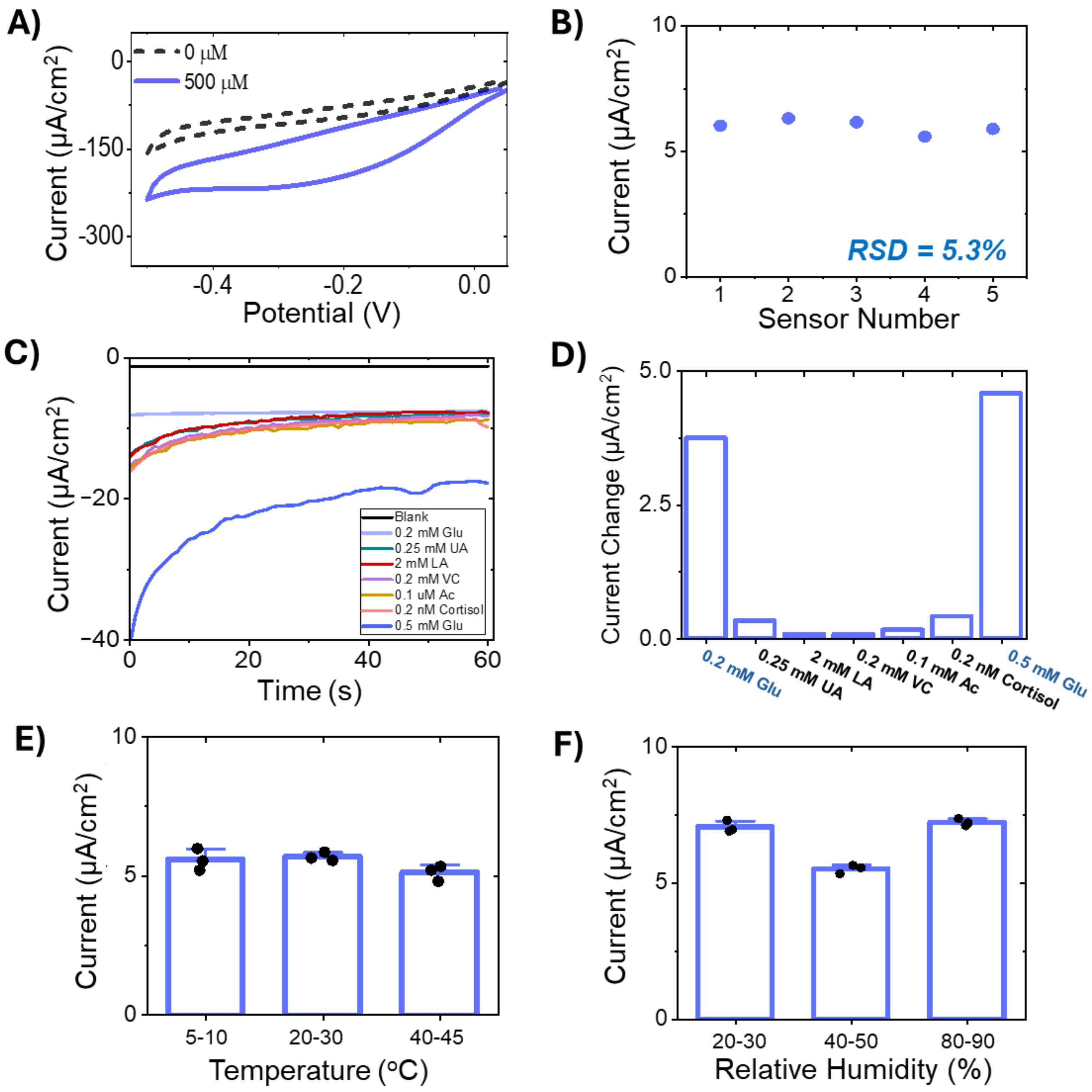

Fig. S13. In-vitro studies of the glucose sensor. A) Cyclic voltammetry plot of the glucose sensor with blank (black dotted) and 500 µM glucose (blue solid). The highest sensitivity was obtained at -0.25V, and this potential was fixed for all chronoamperometric measurements. B) Plot showing the relative standard deviation (RSD) from five different glucose sensors. Such low RSD guarantees reproducible sensor to sensor performance during every measurement. C) Chronoamperometric signals showing the high selectivity of the glucose sensor against other common biomarkers in sweat (Glu-glucose, UA-uric acid, La-lactate, VC-vitamin C, AC -acetaminophen). All added biomarker concentrations match their levels in sweat. Results show the sensor to be highly specific towards glucose.

D) Bar plot summarizing the net current change, as obtained from the selectivity test. E) Plot showing the effect of temperature on the sensor response after 200 μM glucose addition. No significant change was observed. F) Plot showing the small effect of relative humidity on the sensor response after 200 μM glucose addition.

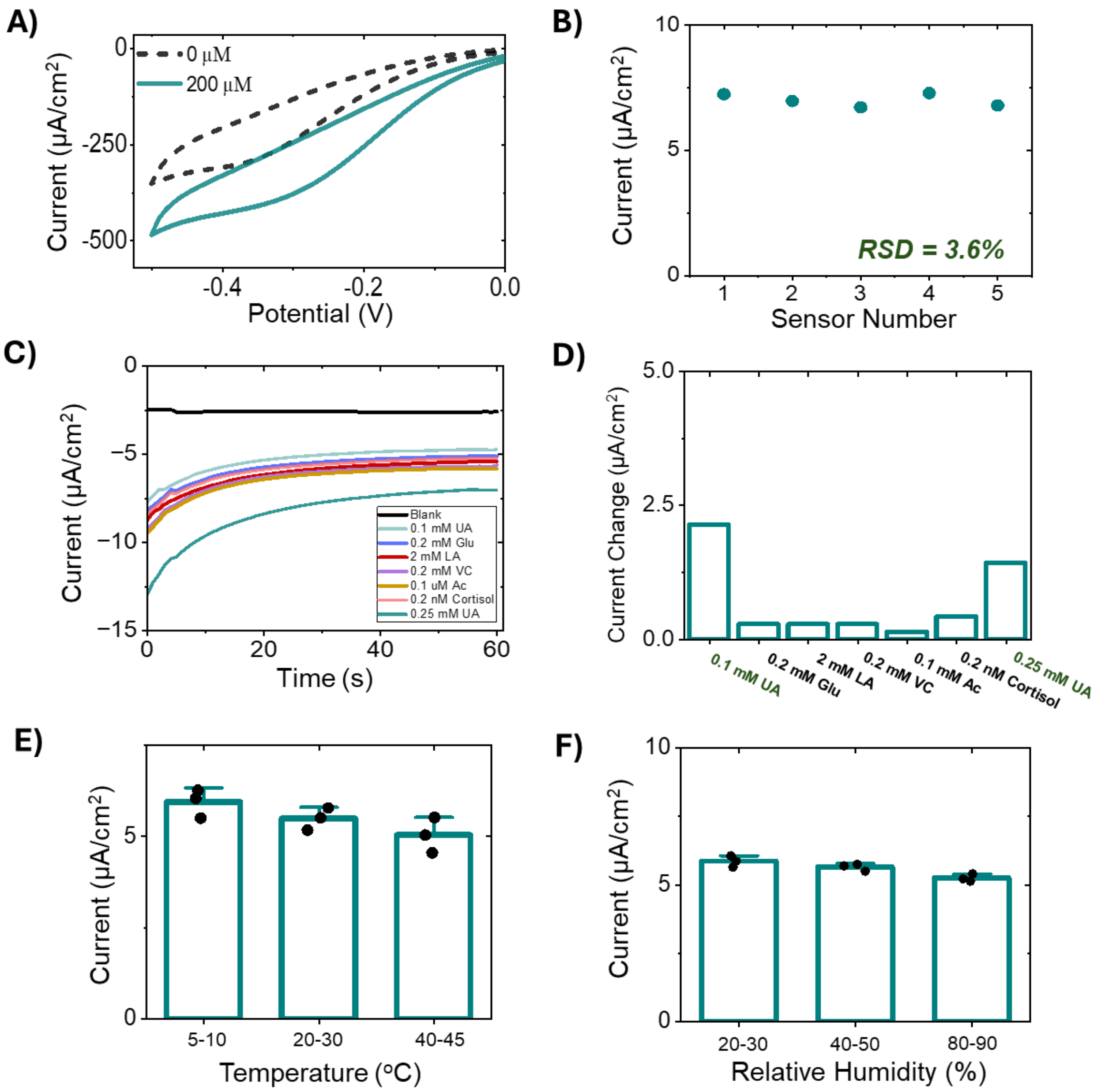

Fig. S14. In-vitro studies of the uric acid (UA) sensor. A) Cyclic voltammograms of the UA sensor with blank (black dotted) and 200 μM UA (green solid). The highest sensitivity was obtained at -0.30V, and this potential was fixed for all chronoamperometric measurements. B) Plot showing the RSD of the response from five different UA sensors. Such low RSD guarantees reproducible sensor-to-sensor performance during each

measurement. C) Chronoamperometric signals illustrating the high selectivity of the UA sensor against other common biomarkers in sweat (UA-uric acid. Glu-glucose, La-lactate, VC-vitamin C, AC -acetaminophen). All added biomarker concentrations match their sweat levels. D) Bar plot summarizing the net current change, as obtained from the selectivity test of C. E) Plot showing the effect of temperature on the response of the UA sensor to 250 μM UA. The UA response at 40-45°C is reduced by ~6% (vs. room temperature), reflecting changes in the uricase activity. F) Plot showing the effect of the relative humidity on the response of the UA sensor to 250 μM UA. No significant change was observed.

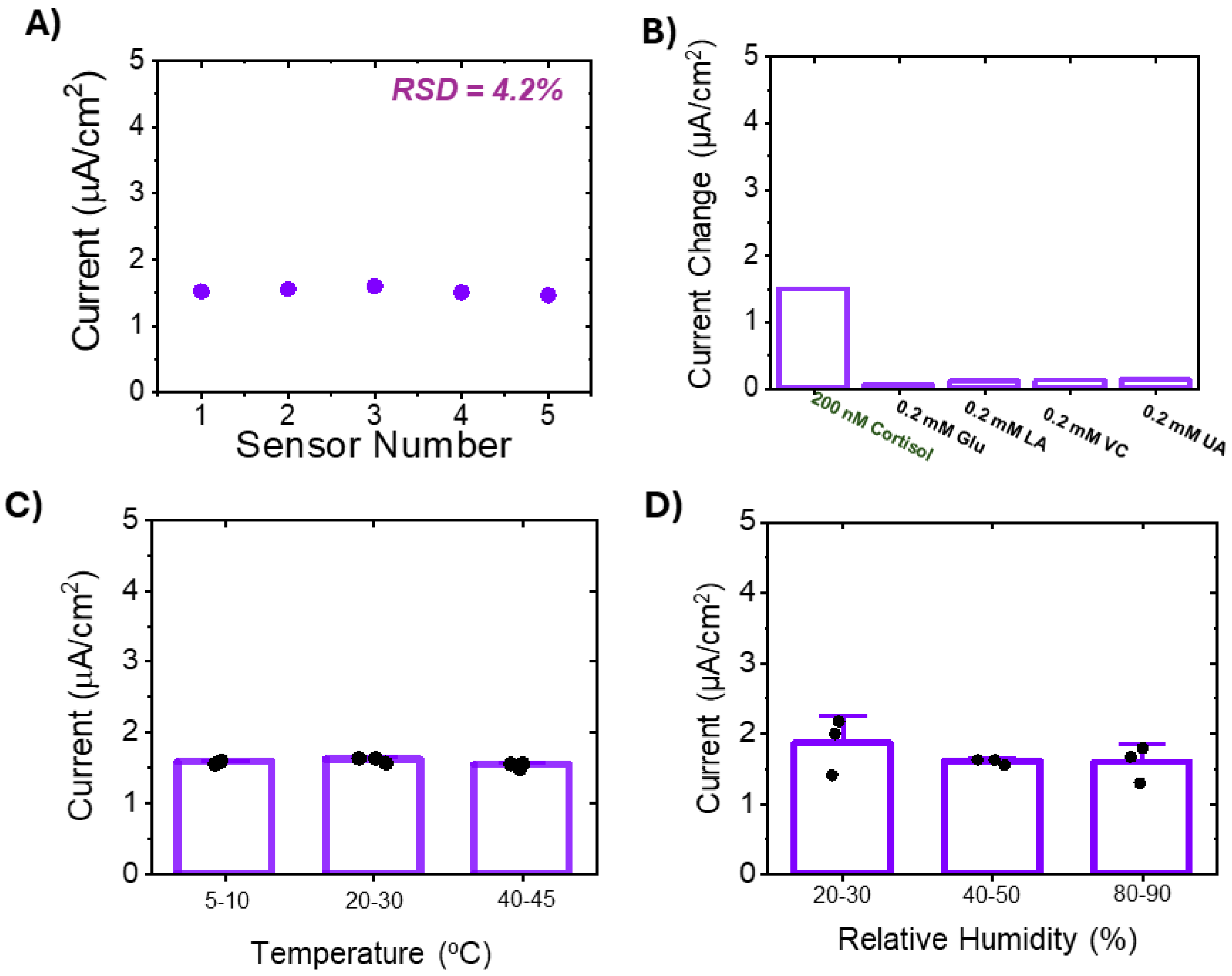

Fig. S15. In-vitro studies of the cortisol sensor. A) Plot showing the RSD of the response from five different cortisol sensors with 200 nM. Such low RSD guarantees reproducible sensor-to-sensor performance during each measurement. B) Bar plot summarizing the net current change from square wave voltammetry with common sweat biomarkers. C) Plot showing the effect of temperature on the response of the cortisol sensor under 200 nM. The response at 40-45°C did not show any significant change vs.

room temperature. D) Plot showing the effect of the relative humidity on the response under 200 nM. No significant change was observed.

Table S3: Comparison of glucose sensor with literature

Ref	Sensor type	Sensitivity	Selectivity	Reproducibility	Detection range	LOD
**This work**	UA-PB	15 μA/mM.cm^2	Stable against glucose, LA, VC, Ac, cortisol	3.6% n=5	Validated till 500 μM	6.2 μM
1	UA-PB	0.04 μA/mM.cm^2	Stable against AA, Ac, alcohol, glucose, LA	4.94% RSD n=8	10-1000 μM	4.95 μM
2	UA-PB	-	Stable against glucose, AA, dopamine, cholesterol	0.53-2.7%, n=6	2.5-400 μM	2.5 μM
3	UA-PB	160 μA/mM.cm^2	Stable against glucose, AA, urea	-	10-160 μM	2.6 μM

1. Moonla, C., Khan, M. I., Akgonullu, S., Saha, T. & Wang, J. Touch-based uric acid sweat biosensor towards personal health and nutrition. *Biosens. Bioelectron.* **277**, 117289 (2025).
2. Jirakunakorn, R. *et al.* Uric acid enzyme biosensor based on a screen-printed electrode coated with Prussian blue and modified with chitosan-graphene composite cryogel. *Microchem. J.* **154**, 104624 (2020).
3. Thakur, B. & Sawant, S. N. Polyaniline/Prussian-Blue-Based Amperometric Biosensor for Detection of Uric Acid. *Chempluschem* **78**, 166–174 (2013).

Table S4: Comparison of UA sensor with literature

Ref	Sensor type	Sensitivity	Selectivity	Reproducibility	Detection range	LOD
**This work**	Pt-Pt	4.2 µA/mM.cm^2	Stable against UA, LA, VC, Ac, cortisol	5.3% n=5	Validated till 1000 µM	11 µM
1	Pt/PtNPs-PANI/SCE	96.1 µA/(mM.cm^2)	Stable against AA, glutathione	-	0.01-8 mM	0.7 µM
2	Pt-Pt-Ag/AgCl	105 µA/(mM.cm^2)	Stable against AA, UA, dopamine	9%, n=3	Till 0.9 mM	10 µM

1. Zhai, D. *et al.* Highly Sensitive Glucose Sensor Based on Pt Nanoparticle/Polyaniline Hydrogel Heterostructures. *ACS Nano* 7, 3540–3546 (2013).
2. Abellán-Llobregat, A. *et al.* A stretchable and screen-printed electrochemical sensor for glucose determination in human perspiration. *Biosens. Bioelectron.* **91**, 885–891 (2017).

Table S5: Comparison of cortisol sensor with literature

Ref	Sensor type	Sensitivity	Selectivity	Reproducibility	Detection range	LOD
**This work**	Au-Au-Ag/AgCl	~ 38 µA/µM	Stable against glucose, LA, VC, UA	4.2%, n=5	Validated till 100 nM	750 pM
1	ITO-C-Ag/AgCl	2 µA/µM	Stable against urea, LA, glucose, testosterone	-	0-5 µM	8 nM
2	Au-Au-Pt	~ 10 µA/µM	-	-	~ 50 nM	-

1. Luan, Y. *et al.* Wearable Sensing Device Integrated with Prestored Reagents for Cortisol Detection in Sweat. *ACS Sensors* 9, 2075–2082 (2024).
2. Yuan, Y., Bali, A., White, R. J. & Heikenfeld, J. Solution-Phase Electrochemical Aptamer-Based Sensors. *IEEE Trans. Biomed. Eng.* **70**, 824–830 (2023).

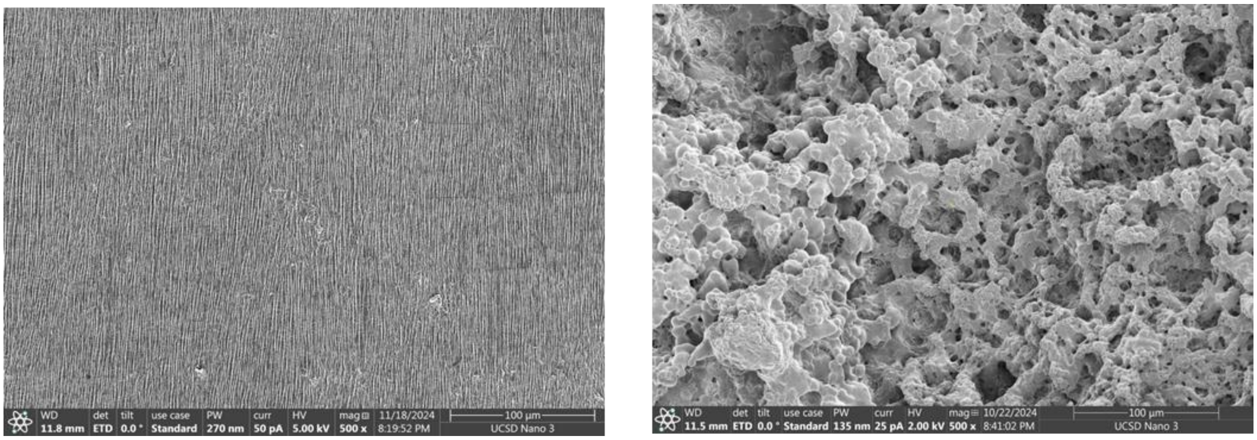

Fig. S16. Scanning electron microscopy (SEM) of hydrogels. SEM images of the non-porous (left) and porous (right) PVA hydrogels. Hydrogel on the right is used for metabolic sensing in TEMPT. A porous gel facilitates sweat transport additionally via Laplace pressure in the capillaries, in addition to just diffusion.

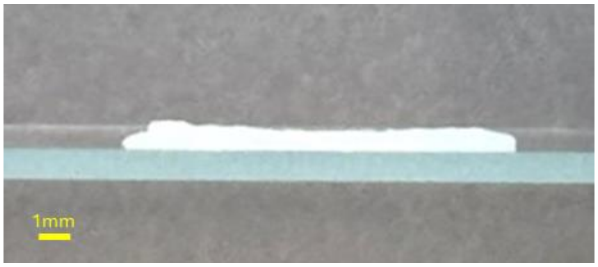

Fig. S17. Estimation of hydrogel thickness. The hydrogel thickness is around 725 µm. Based on this, the diffusion coefficients (D) of the biomarkers were evaluated using:

$$t = \frac{L^2}{2D}$$

where, L is the diffusion path length (hydrogel thickness) and t is the diffusion time (as estimated from Fig. 3j-m).

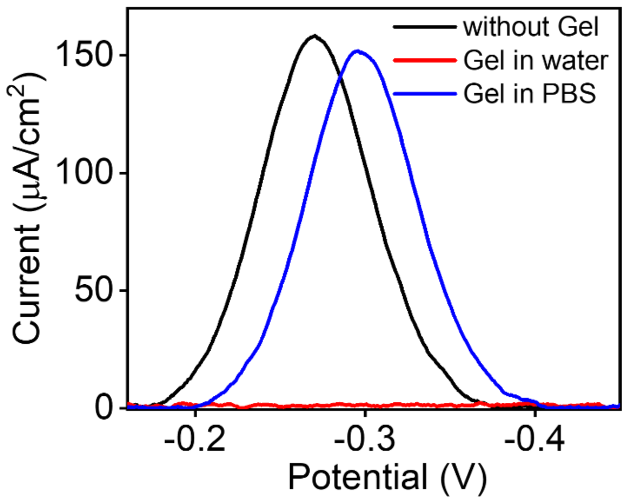

Fig. S18. MB SWV measurements with and without the gel. SWV profiles obtained for cortisol sensor in the absence and presence of gel (soaked in water vs PBS). Results show that aptamers need a conductive media for optimal Operation.

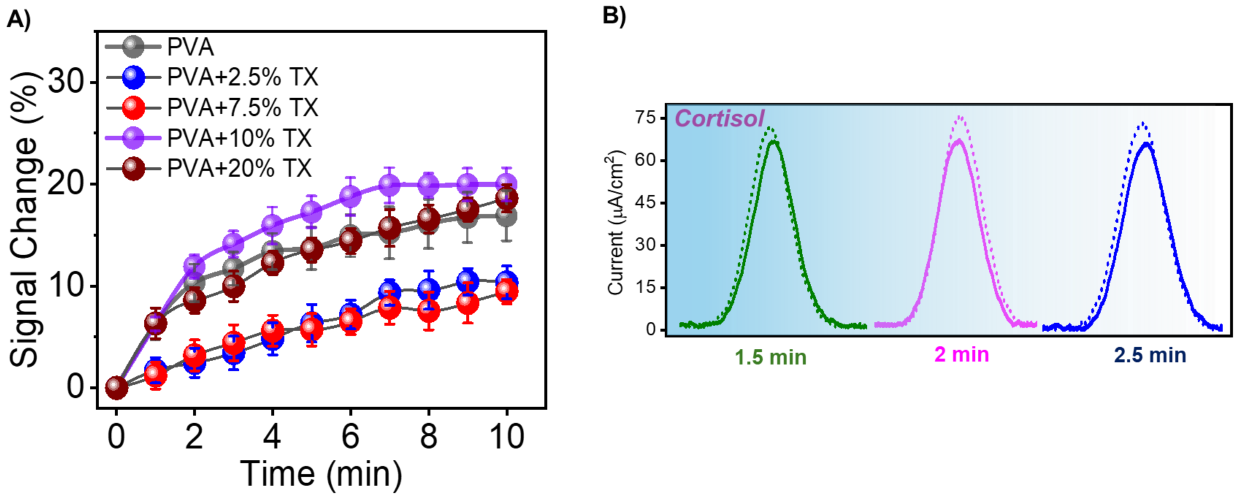

Fig. S19. In-vivo studies of the cortisol sensor. A) Touch-enabled signal changes of cortisol sensor (7 subjects) using different PVA gels treated with TX amounts. PVA+10% TX has been used for all metabolic monitoring. B) Investigation of fingertip touch time duration with MSA and hydrogel using different cortisol sensors. A touch time of at least 2 minutes is needed to transfer all the natural perspiration secreted fingertip cortisol to the hydrogel.

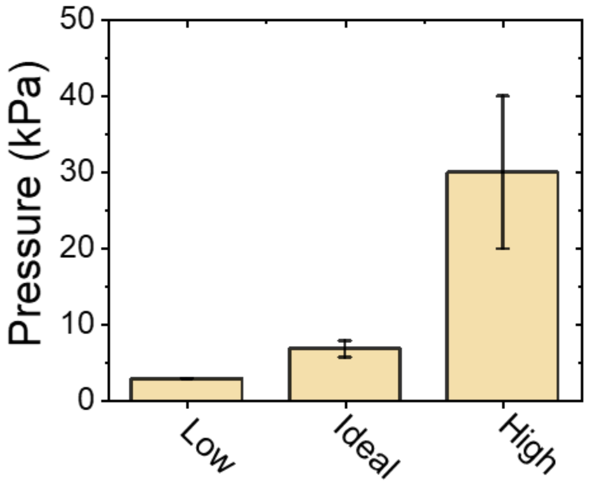

Fig. S20. Pressure analysis of the metabolic touch sensor. Plot categorizing different pressure levels as low, ideal, and high, which are compatible with the touch sensor. Error bar denotes SD from three subjects.

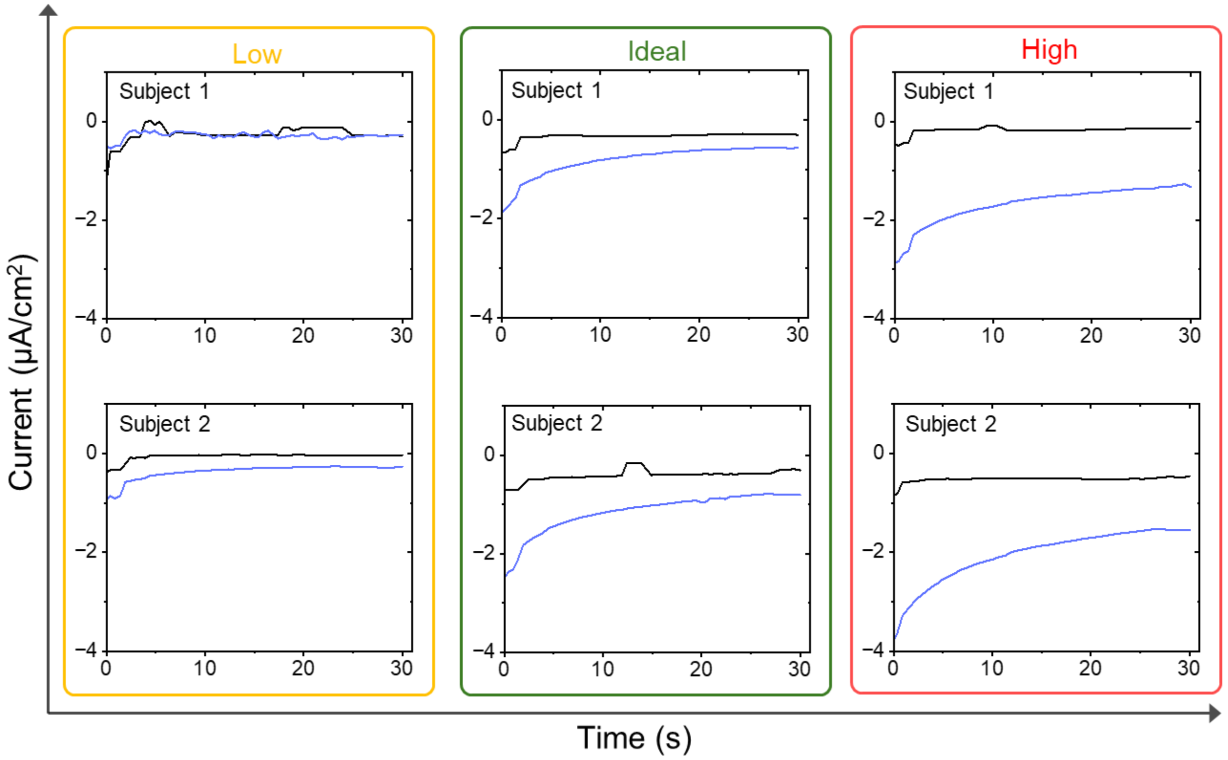

Fig. S21. Effect of touch pressure on the glucose sensor response. Chronoamperometric plot showing the response of 200 μM glucose from two subjects, after touching the glucose sensor (in presence of hydrogel) under varying pressure levels (based on Fig. S12). Each subject applied 5μL's of 200 μM glucose solution on the index fingertip before touching. Results show that excess pressure tends to overestimate the

response. The response from the ideal pressure zone is optimal, as it matches the trend in Fig. 3E.

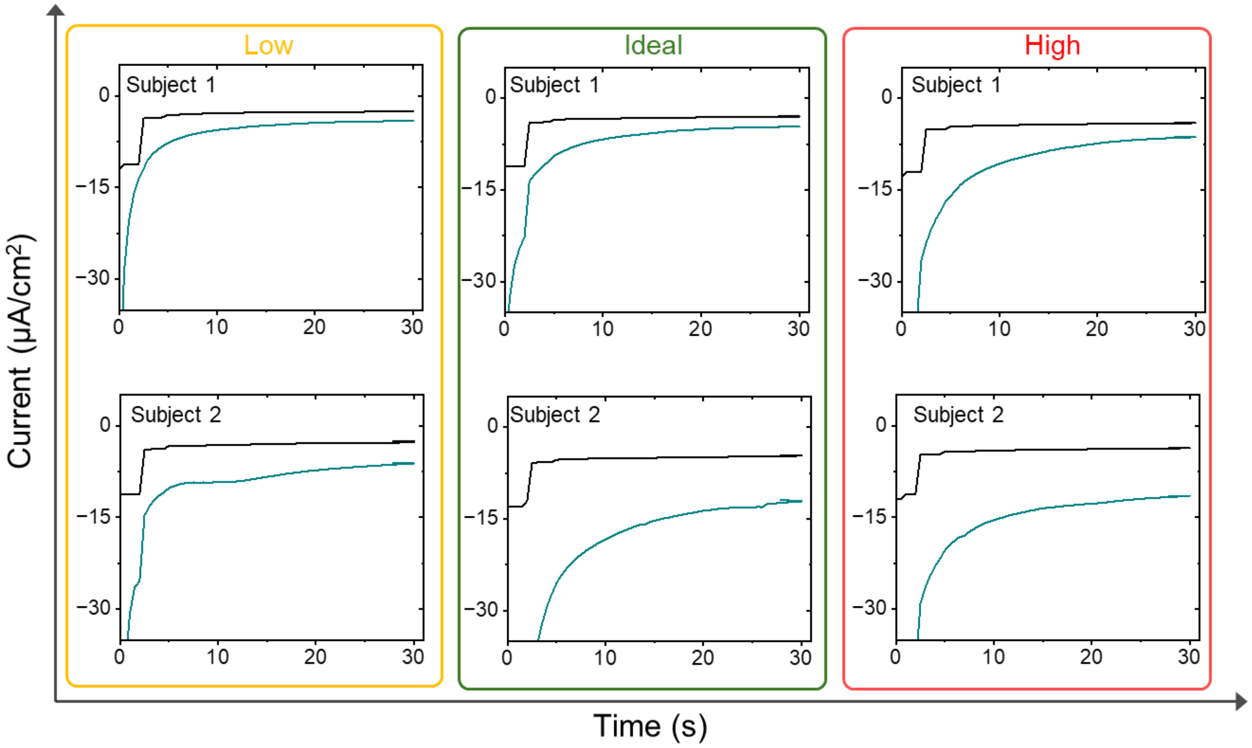

Fig. S22. Effect of touch pressure on the UA sensor response. Chronoamperometric plot showing the response of 250 μM glucose from two subjects, after touching the UA sensor (in presence of hydrogel) under varying pressure levels (based on Fig. S12). Each subject applied 5μL's of 250 μM UA solution on the index fingertip before touching. Results show that excess pressure has a negligible effect on the response (since the response is similar to that in the ideal pressure zone). The responses from both ideal and high-pressure levels also match the response in Fig. 3E.

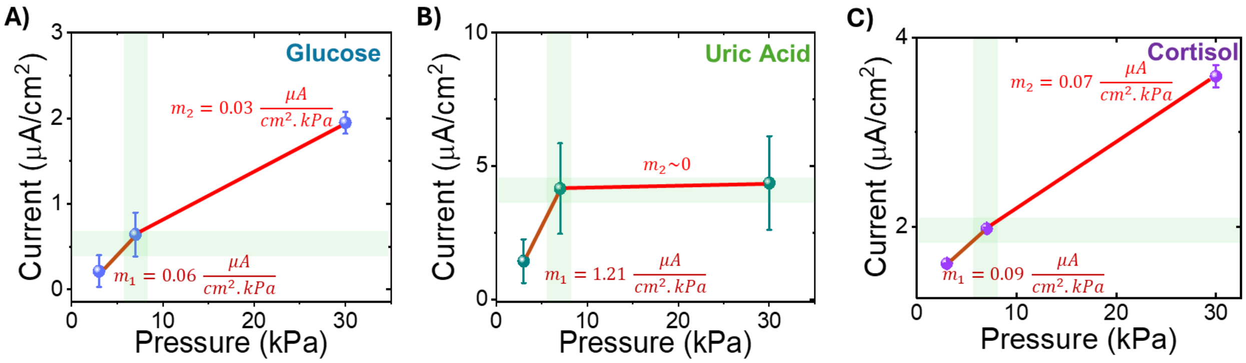

Fig. S23. Effect of touch pressure on the response. A) Effect of touch pressure on the response from 200 μM glucose. The ideal operating pressure ranges ~ 5-6 kPa (green

shade). Under low or high applied pressure, the amperometric signal needs to be corrected. When the applied pressure ranges 7-30 kPa (high), the current change readout needs to be multiplied with 0.03, while it needs to be multiplied by 0.06 under low pressure (2-7 kPa). B) Effect of touch pressure on the response of 250 µM UA. Similar logic also applied to the UA sensor. The reading needs correction (by multiplying 1.21) only under low pressure zone. High pressure zone needs no correction. Brown line: line connecting low and ideal pressures; Red line: line connecting ideal and high pressures. (C) Effect of touch pressure on the response from 200 nM cortisol. The reading needs correction (by multiplying 0.09) under low pressure zone and under high pressure zone (0.07).

Supplementary Text 3: The preparation of the cortisol immunosensor and the detection protocol were followed from our previous study. (1,2) In detail, the thin film sputtered Au electrodes were used for immunoassay experiments. The electrodes were cleaned through the immersion in isopropyl alcohol (IPA) and deionized water.

The sputtered chips containing two Au electrodes, acting as working and counter/reference electrodes, were used for immunosensor preparation. The chips were first immersed in an ethanolic solution of mixed MUA (1 mM)/MCH (10 mM) for overnight to generate the self-assembled monolayer on the electrode surface. After washing the electrodes in ethanol and water, terminal carboxylic groups of the surface thiols were activated by reacting for 35 min with EDC/NHS solution (400/100 mM, prepared in 25 mM MES buffer, pH 6.5). The capture anti-cortisol antibody was immobilized on the Au working electrode through incubating in 100 µg/mL antibody solution for 45 min, followed by washing with PBS. The unreacted carboxylic groups on the electrode were blocked by reacting with 2 M ethanolamine for 30 min. After this, the electrodes were washed with PBS and stored at 4ºC before being used for cortisol detection. The cortisol detection was performed through a competitive immunoassay format, where 1:50 dilution of HRP-tagged cortisol was mixed with various concentrations of cortisol antigen within the range of 1 nM to 1000 nM in artificial sweat solution. The competitive reaction was performed for 15 min, after which the electrodes were subsequently washed with PBS containing 0.05% SDS and the PBS, respectively and dried. The amperometric transduction was performed immediately after covering the electrode chip with 25 µL of TMB/H_2O_2 reagent at applied potential of -0.1 V for 90 s.

1) E. Vargas, E. Povedano, S. Krishnan, H. Teymourian, F. Tehrani, S. Campuzano, E. Dassau, J. Wang, Biosensors and Bioelectronics 2020, 167, 112512.

2) W. Tang, L. Yin, J. R. Sempionatto, J. Moon, H. Teymourian, J. Wang, Adv. Mater. 2021, 33, 2008465.

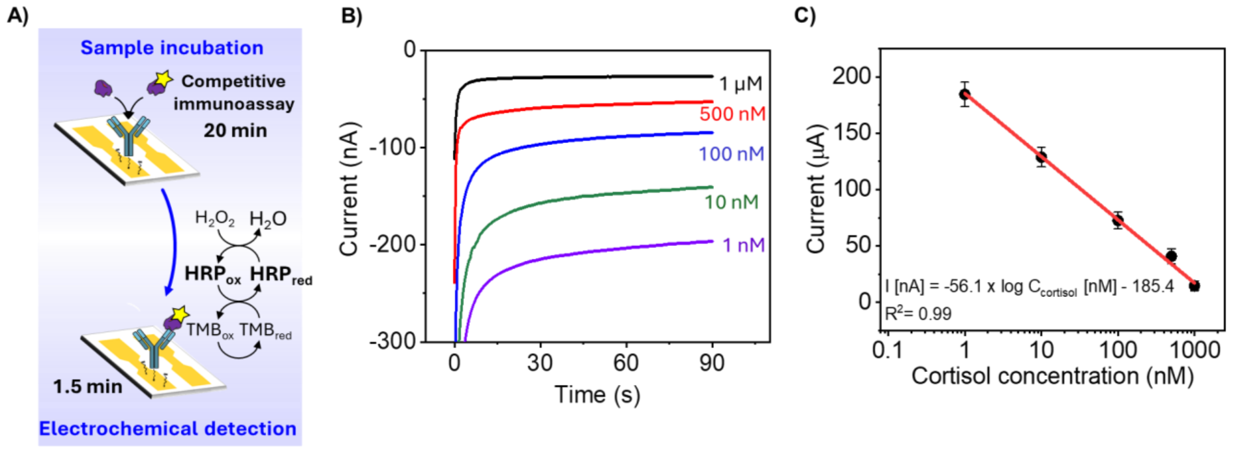

Fig S24. Cortisol immunosensor calibration. A) Schematic highlighting the working principle of the cortisol competitive immunoassay. (B) Chronoamperometric response at -0.1 V for different cortisol concentrations with the (C) corresponding cortisol immunosensor calibration curve.

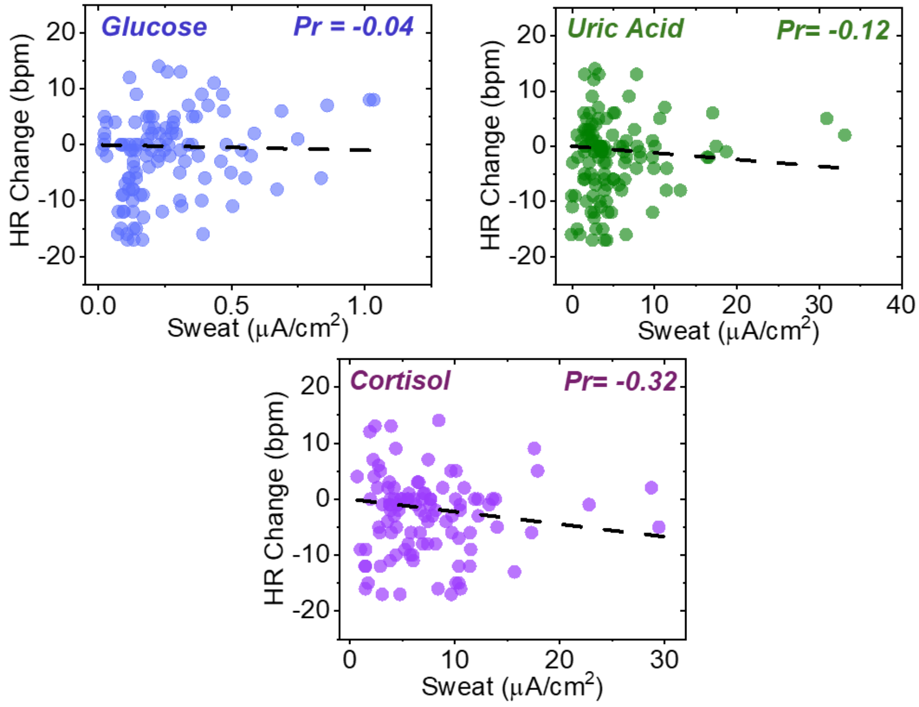

Fig. S25. Effect of sweat biomarkers on heart rate. Plots showing the correlation of HR with sweat biomarkers. HR change shows the highest correlation to sweat glucose change (n=110), followed by uric acid (n=120) and cortisol (n=110). This is because glucose, UA, and HR change frequently with meals, which have been included multiple times in the extended studies. Cortisol and HR together change only with CPT and walk, which have been introduced only a few times in the extended studies. Hence, HR change does not hold a significant relevance (vs. MAP change) to the sweat biomarker levels.
